# Select amino acids recover cytokine-altered ENaC function in human bronchial epithelial cells

**DOI:** 10.1371/journal.pone.0307809

**Published:** 2024-07-25

**Authors:** Anusree Sasidharan, Astrid Grosche, Xiaodong Xu, T. Bernard Kinane, Damiano Angoli, Sadasivan Vidyasagar

**Affiliations:** 1 Department of Radiation Oncology, Shands Cancer Center, University of Florida, Gainesville, Florida, United States of America; 2 Pediatric Pulmonary Division, Massachusetts General Hospital for Children, Boston, Massachusetts, United States of America; 3 Harvard Medical School, Boston, Massachusetts, United States of America; 4 Pediatric Pulmonary Division, College of Medicine, University of Florida, Gainesville, Florida, United States of America; China Medical University, TAIWAN

## Abstract

The airway epithelium plays a pivotal role in regulating mucosal immunity and inflammation. Epithelial barrier function, homeostasis of luminal fluid, and mucociliary clearance are major components of mucosal defense mechanisms. The epithelial sodium channel (ENaC) is one of the key players in controlling airway fluid volume and composition, and characteristic cytokines cause ENaC and barrier dysfunctions following pulmonary infections or allergic reactions. Given the limited understanding of the requisite duration and magnitude of cytokines to affect ENaC and barrier function, available treatment options for restoring normal ENaC activity are limited. Previous studies have demonstrated that distinct amino acids can modulate epithelial ion channel activities and barrier function in intestines and airways. Here, we have investigated the time- and concentration-dependent effect of representative cytokines for Th1- (IFN-γ and TNF-α), Th2- (IL-4 and IL-13), and Treg-mediated (TGF-β1) immune responses on ENaC activity and barrier function in human bronchial epithelial cells. When cells were exposed to Th1 and Treg cytokines, ENaC activity decreased gradually while barrier function remained largely unaffected. In contrast, Th2 cytokines had an immediate and profound inhibitory effect on ENaC activity that was subsequently followed by epithelial barrier disruption. These functional changes were associated with decreased membrane protein expression of α-, β-, and γ-ENaC, and decreased mRNA levels of β- and γ-ENaC. A proprietary blend of amino acids was developed based on their ability to prevent Th2 cytokine-induced ENaC dysfunction. Exposure to the select amino acids reversed the inhibitory effect of IL-13 on ENaC activity by increasing mRNA levels of β- and γ-ENaC, and protein expression of γ-ENaC. This study indicates the beneficial effect of select amino acids on ENaC activity in an *in vitro* setting of Th2-mediated inflammation suggesting these amino acids as a novel therapeutic approach for correcting this condition.

## Introduction

Specialized epithelial cells lining the respiratory tract from the nasal cavity to the alveoli represent the first line of defense against inhaled gases, microorganisms, or other particulate matter. They accomplish this by maintaining extracellular fluid homeostasis along airway surfaces through various mechanisms such as epithelial ion transport, barrier function, and regulated mucus production. Inflammation has the potential to disrupt these mechanisms, leading to airway surface dehydration or abnormal airway secretions [1]. The airway epithelium is covered by a thin layer of periciliary fluid (~7 μm) and a luminal mucus layer that is well-hydrated (~7–70 μm) collectively referred to as airway surface liquid (ASL). ASL plays a key role in maintaining a physical barrier facilitating defense mechanisms by capturing, neutralizing, and/or removing inhaled gases, microorganisms, and other particles through mucociliary clearance [2, 3]. The volume and composition of ASL are precisely regulated through transepithelial ion and water transport, orchestrated by a complex interplay between apical anion secretion via anion channels and sodium reabsorption via a high-affinity sodium-selective pore, the epithelial sodium channel (ENaC) [2, 4]. In addition, the epithelial barrier function modulates permeability through selective pores within the tight junction complex [5].

Reabsorption of sodium through ENaC is a major element in the regulation of extracellular fluid, and disruptions in ENaC function have been associated with various airway diseases, including cystic fibrosis, allergic rhinitis, asthma, pulmonary edema, and acute lung injury [6]. Considering the need for rapid and dynamic adjustments in ion and water movement, ENaC activity is meticulously controlled by a myriad of factors, including circulating hormones, metabolic alterations or inflammatory stimuli that affect cytoskeleton, proteolytic cleavage, trafficking, membrane stability, and proteasomal degradation [7–12]. In humans, ENaC consists of four homologous subunits α, β, γ and δ, arranged in a configuration of α-β-γ or δ-β-γ. The δ subunit is not expressed in mice and rats [13]. The expression of three subunits is required for full channel activity, although the α-subunit alone, or a combination of αβ or αγ can also generate small sodium currents [14].

Changes in ENaC function are frequently observed in cell-mediated (Th1) inflammatory scenarios that are usually associated with the production of pro-inflammatory cytokines, including TNF-α and IFN-γ, and in immune responses that drive humoral (antibody) immunity (Th2-mediated), where IL-4 and/or IL-13 predominate [4]. IFN-γ, a pro-inflammatory cytokine released by T-lymphocytes during viral infections, has been shown to reduce ENaC activity. In contrast, TNF-α released by macrophages and neutrophils during acute inflammation has no effect on sodium absorption in bronchial epithelial cells but decreased α- and γ-ENaC mRNA, and protein levels [4, 15–17]. Cytokines released during antiviral immune responses have also been shown to increase epithelial permeability by decreasing the expression levels of claudins, occludin, and ZO-1 [18–22]. Increased production of IL-4 and/or IL-13, which is associated with allergic airway diseases, usually causes goblet cell hyperplasia and mucus secretion. However, studies on airway epithelial cells have demonstrated that these cytokines also alter ion transport and barrier function. Treatment with IL-4 or IL-13 reduced amiloride-sensitive short-circuit currents by decreasing the expression levels of ENaC subunits γ and β in bronchial epithelium, and by decreasing occludin and claudin-3 protein expression in nasal epithelial cells [23–26].

Interestingly, TGF-β1, a pleiotropic cytokine produced by regulatory T cells (Treg) that modulates the immune system, maintains tolerance to self-antigen and prevents autoimmune diseases, decreases ENaC activity by reducing mRNA and protein expression of the α subunit and by β subunit internalization [27–29]. TGF-β1 which plays a crucial role in both, Th1 and Th2 immune responses, is a critical mediator of acute lung injury.

Although, disrupted sodium absorption via ENaC and/or increased permeability in airway epithelium contribute to the pathogenesis of various Th1- or Th2-mediated inflammatory lung diseases, little is known about the progression and significance of changes in ENaC and barrier function in the presence of these cytokines over time [23, 30, 31]. Moreover, there are currently no therapeutic agents approved that can recover ENaC activity in affected patients. In this study, normal primary human bronchial epithelial cells (HBECs) were exposed to representative cytokines and combinations thereof that simulate Th1- and Th2-mediated immune responses, and their effect on ENaC activity and barrier function was evaluated in a dose- and time-dependent manner. In prior studies, it was demonstrated that certain amino acids regulate ion channel and barrier function by modulating the expression and activity of proteins responsible for these functions in both mouse intestine and HBECs [32–34]. To explore the potential for an amino acid formulation to help recover ENaC activity in characteristic inflammatory settings, we tested the formulation “AA-T” for its ability to regulate ENaC function in an *in vitro* model of Th1- (TNF-α, IFN-γ, and TGF-β1) and Th2-mediated inflammation (IL-4 and IL-13) in primary HBECs. By its ability to recover ENaC activity, the amino acid test formulation may serve as a singular therapeutic approach for multiple causal factors where the lack of airway fluid absorption can be triggered.

## Material and methods

### Human bronchial epithelial cell culture

Primary HBECs were obtained from the University of Alabama CF Research and Translation Core Center under a Material Transfer Agreement with the University of Florida (UAB A-302178). Because the cells were obtained from deceased individuals with minor, de-identified information, its use does not constitute human subjects research as defined by CFR 46.102. The provider assured that the cells were acquired in accordance with the Common Rule, and the project was approved by the Institutional Review Board of the University of Alabama (IRB 00000726). A written consent was obtained before procurement of the cells. All cell culture experiments were performed in agreement with the guidelines and regulations described by the Declaration of Helsinki and the Huriet-Serusclat and Jardet law governing human research ethics. The protocols to acquire, culture, store, and study HBECs were approved by the Institutional Review Board of the University of Florida (IRB 201700635).

Normal primary HBECs at passages 2 and 3, recovered from four non-smoker lung donors were seeded on permeable snapwell inserts coated with human placenta-derived collagen IV (Sigma, USA) for Ussing chamber studies, or on transwell inserts for western blot and real-time quantitative polymerase chain reaction (real-time qPCR) with 0.4 μM pore polycarbonate membrane (Corning Costar, USA). The cells were seeded at 8 x 10^5^ cells·cm^-2^ in an expansion medium (PneumaCult Ex Plus; StemCell Technologies, USA) containing 1% penicillin/streptomycin, and incubated at 37°C and 5% CO_2_. After expansion to 95% confluence (cells were submerged in expansion medium), cells were differentiated in PneumaCult^TM^ ALI medium (StemCell Technologies, USA) containing 1% penicillin/streptomycin at an air-liquid interface. ALI medium was changed every other day until cells were fully differentiated (14–21 days). Differentiated HBECs are characterized by the presence of active ciliary motility. Age-matching HBECs were randomly assigned to the treatment groups for subsequent experimentation.

### Cytokine treatments

Human recombinant cytokines (IL-13, IL-4, TNF-α, IFN-γ, and TGF-β1) were added to the ALI medium, starting 14 days after cells were air-lifted. The cell culture medium containing cytokines was changed every other day. For dose-dependent studies, IFN-γ or TNF-α (R&D Systems, USA) were used at concentrations of 5x10^-5^, 5x10^-4^, 5x10^-3^, 5x10^-2^, 0.5, 5, 10, 20, 40, and 50 ng/mL, while TGF-β1 (R&D Systems, USA) was used at concentrations of 5x10^-5^, 5x10^-4^, 5x10^-3^, 5x10^-2^, 0.5, 5, and 50 ng/mL for 7 days. IL-13 (R&D Systems, USA) was used at concentrations of 0.1, 0.2, 0.5, 1, 2, 4, 8, 16, 20, and 64 ng/mL for 14 days. Time-dependent studies were performed at concentrations that ensured maximal inhibition of benzamil-sensitive *I*_sc_ and TEER. Specifically, HBECs were treated with IFN-γ, TNF-α, or TGF-β1 at 1 ng/mL, IL-13 at 20 ng/mL, and IL-4 (PeproTech, USA) at 2 ng/mL for 2, 4, 6, 8, 10, 12, 14, and 16 days.

A cytokine cocktail containing IFN-γ and TNF-α was used at concentrations of 0.05, 0.5, 2.5, 5, and 10 ng/mL for each cytokine. Both cytokines were added to the culture medium for 7 days. All samples were pooled for subsequent statistical analysis, and no data outliers were excluded.

### Test formulations

Ringer (CON), amino acid test formulation (AA-T), and negative control amino acids (AA-NC) were prepared as equimolar solutions and used for Ussing chamber studies, patch clamp recordings, western blot analyses, real-time qPCR, and immunofluorescence experiments. The Ringer solution contained (in mmol/L) 113.8 Na^+^, 93.6 Cl^-^, 25 HCO_3_^-^, 5.2 K^+^, 2.4 HPO_4_, 0.4 H_2_PO_4_^-^, 1.2 Mg^2+^, 1.2 Ca^2+^, and 75 mannitol adjusted to pH 7.4 and 300mOsm. AA-T contained 8 mmol/L L-lysine, L-tryptophan, L-arginine, and L-glutamine, and 1.2 mmol/L L-tyrosine. AA-NC contained 8 mmol/L L-leucine, L-cysteine, L-isoleucine, L-aspartic acid, and L-glutamate (Ajinomoto, USA), dissolved in a modified Ringer solution containing (in mmol/L) 113.8 Na^+^, 93.6 Cl^-^, 25 HCO_3_^-^, 5.2 K^+^, 2.4 HPO_4_^-^, 0.4 H_2_PO_4_^-^, 1.2 Mg^2+^, 1.2 Ca^2+^, and 40 mannitol at pH 7.4 to match the osmolality of 300 mOsm in Ringer solution.

### Ussing chamber studies

Snapwells with differentiated cytokine-treated or untreated HBECs were mounted in Ussing chambers (VCC MC8; Physiologic Instruments, USA) that were pre-warmed to 37°C and calibrated in Ringer solution. Cells were either bathed in iso-osmolar Ringer solution, AA-T, or AA-NC. Additionally, glucose (5 mmol/L) was added to the basolateral side of the Ussing chambers, and chambers were bubbled with 95% O_2_ and 5% CO_2_ and maintained at 37°C. Inserts with HBEC cultures were allowed to equilibrate in Ussing chambers for 30 minutes, and transepithelial short-circuit current (*I*_sc_) and transepithelial electrical resistance (TEER) were measured across the HBEC layer using standard Ohm’s law. To eliminate the influence of paracellular ion movements driven by passive forces such as transepithelial concentration gradients, osmotic gradients, and hydrostatic gradients, the membrane potential was continuously clamped to zero by applying an external current across the epithelium. This external current is recorded as *I*_sc_ and is equivalent to the algebraic sum of the electrogenic ion movement by active transport across the epithelium. Basal *I*_sc_ and TEER were recorded at 30-second intervals. TEER > 150 Ω·cm^-2^ was considered adequate for assessing the integrity of HBEC cultures. Benzamil-sensitive *I*_sc_ was subsequently calculated as the difference between the basal *I*_sc_ measured at 30 minutes and *I*_sc_ measured 15 minutes after the addition of 6 μM benzamil (Tocris, USA) to the apical side.

### Patch clamp analysis

For the patch clamp recordings, differentiated HBECs were trypsinized and seeded on collagen IV-coated glass coverslips in ALI medium. The cells were maintained for at least 24 to 48 hours before electrophysiological recordings. The coverslips were mounted on RC-21BDW (Warner Instruments, USA) and viewed using an inverted microscope. The internal pipette solution contained (in mmol/L): 20 KCl, 120 K-Gluconate, 5 HEPES, 0.2 EGTA, and 0.5 MgCl_2_. The bath Ringer solution contained (mmol/L): 113.8 Na^+^, 93.6 Cl^-^, 25 HCO_3_^-^, 5.2 K^+^, 2.4 HPO_4_^-^, 0.4 H_2_PO_4_^-^, 1.2 Mg^2+^, 1.2 Ca^2+^, and 75 mannitol. Since gassing cells in our patch clamp setup was not possible, steps were undertaken to show that the pH was stable for up to 3 hours when 25 mM HCO_3_^—^containing buffer previously bubbled with 5% CO_2_ and pH adjusted to 7.4 was exposed to air. All our whole-cell recordings were under 2 hours and therefore supported the use of 25mM HCO_3_^—^containing buffer for patch clamp experiments without bubbling with 5% CO_2_. The Ringer and amino acid solutions were freshly prepared and bubbled with 95% O_2_ and 5% CO_2_ before each experiment to ensure a stable pH of 7.4 during the recordings. Three sets of recordings were performed utilizing a ramp voltage clamp protocol spanning from -80 mV to +80 mV with a holding potential of -10 mV to evaluate the amount of ENaC current blocked by benzamil (6 μM). The blocking effect of benzamil on ENaC activity was initially recorded in untreated cells, serving as controls. Subsequently, the same protocol was used in two sets of recordings performed on HBECs treated with IL-13 for a minimum of 4 days, and IL-13-treated cells exposed to AA-T for 15 minutes. To prevent amino acid-coupled sodium currents during whole-cell recordings, the cells were washed with Ringer solution for another 15 minutes before further recordings.

### Immunofluorescence

For immunofluorescence, untreated control and IL-13-treated HBECs (20 ng/ml; 14 days) were exposed to either Ringer solution (CON), AA-T, or AA-NC (200 μL per filter) on the apical side of the culture inserts and incubated for 1 hour at 37°C and 5% CO_2_. Following exposure, the cells were fixed in 4% paraformaldehyde and embedded in paraffin. Paraffin sections of 4 μm thickness were mounted on silane-coated glass slides, deparaffinized, rehydrated, and heat pre-treated in retrieval buffer at pH 6.0 (Biocare Medical, USA) as previously described [32]. After blocking with 1% BSA and 10% normal goat serum, sections were incubated in rabbit anti-human SCNN1A (Antibodies-Online, USA; Cat# ABIN863204; RRID: AB_2921336), SCNN1B (ThermoFisher, USA; Cat# PA5-76060; AB_2719788), or SCNN1G polyclonal antibody (StressMarq, USA; Cat# SPC-405; RRID: AB_10640369) diluted in blocking buffer (1:100) overnight at 4°C. Goat-anti-rabbit superclonal recombinant secondary antibody conjugated with Alexa Fluor 647 (ThermoFisher, USA; Cat# A27040; RRID: AB_2536101) was used at a concentration of 1 μg/mL and incubated for 1 hour. Nuclei were stained with DAPI for 15 minutes, and cells were mounted in an aqueous mounting medium (Abcam, USA) before analysis. Signals were analyzed at 600X magnification using the Laser Scanning confocal microscope (Fluoview FV1000, Olympus, Japan).

### Western blotting

Differentiated control and IL-13-treated HBECs (20 ng/mL; 10 days) grown on 24 mm transwell filters (Corning Costar, USA) were exposed to 1 mL of Ringer solution (CON), AA-T, or AA-NC on the apical side for 1 hour at 37°C and 5% CO_2_. After removing the treatment formulations, cells were incubated in 3 mmol/L dithiothreitol (DTT) diluted in PBS for 30 minutes. Cells were then washed three times with ice-cold PBS and harvested from the inserts using a cell scraper. The cell suspension was centrifuged at 1200 x g for 5 minutes, and membrane proteins were isolated from the cell pellet using the “Minute Plasma Membrane Protein Isolation and Cell Fractionation Kit” (Invent Biotech, USA) following the manufacturer’s instructions. The protein concentration in each extract was determined by BCA assay (Sigma, USA). For α-ENaC and β-ENaC, 20 μg of total membrane protein, and γ-ENaC, 10 μg of total membrane protein were subjected to sodium dodecyl sulfate-polyacrylamide gel electrophoresis. Proteins were transferred to polyvinylidene difluoride membranes and probed with primary polyclonal rabbit-anti-human α-ENaC (SCNN1A; ThermoFisher, USA; Cat# PA1-920A; RRID: AB_2184369), β-ENaC (SCNN1B; Invitrogen, USA; Cat# PA5-76060; RRID: AB_2719788), γ-ENaC (SCNN1G; Abcam, USA; Cat# ab3468: RRID: AB_303829), and β-actin (Proteintech, USA; Cat# CL594-66009; RRID: AB_2883475). The membranes were incubated with corresponding secondary antibodies (IRDye 800CW for the protein of interest, and IRDye 680 CW for β-actin; LI-COR Biosciences, USA). Signal detection was performed using Odyssey CLX (LI-COR Biosciences, USA). The abundance of the protein of interest was normalized to β-actin and expressed as relative fold changes.

### Real-time qPCR

For real-time qPCR experiments, differentiated HBECs grown on 12 mm transwell filters (Corning Costar, USA) were treated with 20 ng/mL of IL-13 for 7 days. Commencing on day 3 of this treatment, cells were additionally exposed to 50 μL of either Ringer solution (CON), AA-T, or AA-NC which was added daily to the apical side of the cell cultures for the remaining 5 days of IL-13 treatment. On day 7, the final exposure to Ringer, AA-T, or AA-NC (200 μL per filter) was carried out for 3 hours at 37°C and 5% CO_2_ to simulate the experimental conditions in Ussing chambers. After removing the formulations, the cells were incubated in 3 mmol/L DTT diluted in PBS for 30 minutes at room temperature. Subsequently, the cells were washed three times with ice-cold PBS and harvested from the inserts using a cell scraper. The suspended cells were centrifuged at 1200 x g for 5 minutes. For further processing, lysis buffer from the RNeasy Mini Kit (Qiagen, USA) was added to the cell pellet, and the cell suspension was vortexed for 2 minutes as per the manufacturer’s instructions. The mRNA concentration was estimated spectrophotometrically using the Nanodrop method (Agilent BioTek Epoch, USA), and cDNA was synthesized using an iScript cDNA Synthesis Kit (BioRad, USA). Quantitative PCR reactions were performed in duplicates with two independent experiments for each donor. SsoAdvanced Universal SYBR Green Supermix (Bio-Rad, USA), and the primer sets listed in Table 1 were used at their optimum melting and annealing temperatures in a CFX-Connect Real-Time PCR detection system (Bio-Rad, USA). Relative quantification of mRNA expression was calculated using the Pfaffl method [35] with RPS13 as the reference gene.

**Table 1:** Real-time qPCR primers.

Gene	Protein	Forward primer	Reverse Primer
*hSCNN1A*	α-ENaC	CAACAACGGTCTGTCCCTGA	CCGCAAGTTAAAGCCACCAT
*hSCNN1B*	β-ENaC	ATATCACCCTGAGCAGGAAGGG	CCAGACGATGTTATTGGCTGCT
*hSCNN1G*	γ-ENaC	AGCCCAGCCAACAGTATTGAGA	GACAACAGAGCAGCTCATCCAC
*hRPS13*	RPS13	CGAAAGCATCTTGAGAGGAACA	TCGAGCCAAACGGTGAAT

### Statistical analysis

Results are presented as mean ± standard error of the mean (SEM). Statistical analyses were performed with the OriginPro 2020 software package. For each treatment group, values were tested for normal distribution using the Shapiro-Wilk normality test. The Kruskal-Wallis test was used to compare the overall effect of the cytokine concentrations and treatment duration as well as the effect of Ringer, AA-T, and AA-NC on mRNA levels, and membrane protein expression. Whenever a significant P value was identified, the Mann-Whitney U test was used for comparison between basal values for each cytokine at 0 ng/mL or on day zero with each concentration or time period studied, and for pairwise comparisons of mRNA and protein values between the treatment groups. The effect of Ringer, AA-T, or AA-NC on benzamil-sensitive *I*_sc_, and TEER was calculated with one-way ANOVA followed by post hoc Bonferroni test for individual comparison. For patch clamp whole-cell recordings, a paired t-test was applied to analyze the response of benzamil on current changes in comparison to basal values, and one-way ANOVA followed by Bonferroni test was performed to calculate differences between the treatment groups. A P value of < 0.05 was considered significant for all analyses.

## Results

### IFN-γ affects epithelial barrier function over time, without significantly impacting ENaC activity, while TNF-α disrupts ENaC activity at low concentrations

**Fig 1:**
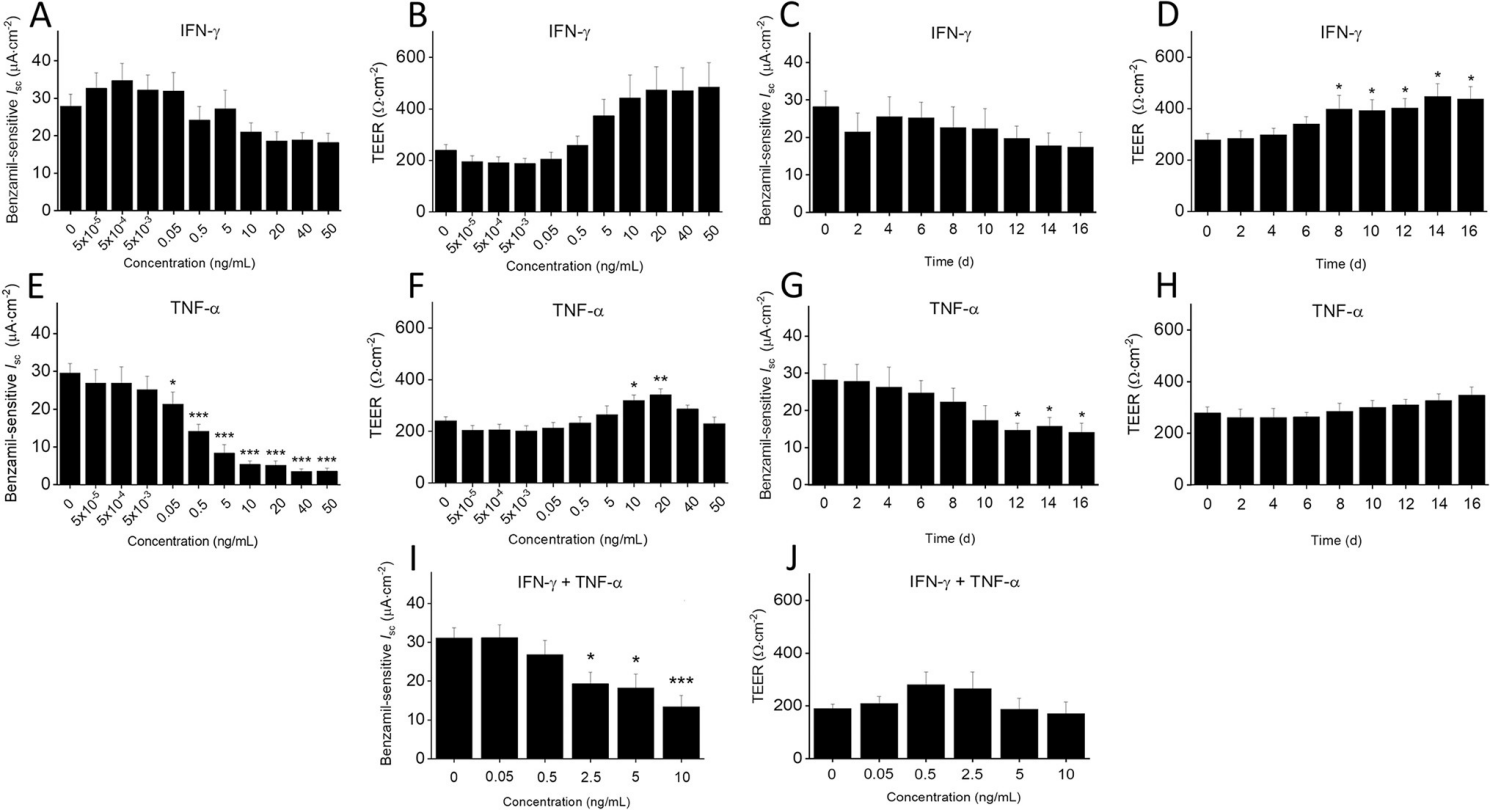
Dose- and time-dependent effect of IFN-γ and TNF-α on benzamil-sensitive *I*_sc_ and TEER in HBECs. Dose-dependent effect of the cytokines on benzamil-sensitive *I*_sc_ and TEER was analyzed in primary, fully differentiated HBECs that were incubated with increasing concentrations of IFN-γ or TNF- α (5 x 10^−5^ to 50 ng/mL) for 7 days. The time-dependent effect of the cytokines on benzamil-sensitive *I*_sc_ and TEER was analyzed in HBECs that were incubated with IFN-γ or TNF-α at 1 ng/mL for 16 days, and data were recorded on days 0, 2, 4, 6, 8, 10, 12, 14, and 16. Delta *I*_sc_ was calculated from *I*_sc_ before and 15 minutes after adding 6 μM benzamil apically to the Ussing chamber. TEER was recorded 30 minutes after mounting the cell culture inserts in Ussing chambers. **(A)** Dose-dependent effect of IFN-γ on benzamil-sensitive *I*_sc_ after exposure for 7 days. **(B)** Dose-dependent effect of IFN-γ on TEER after exposure for 7 days. **(C)** Time-dependent effect of IFN-γ on benzamil-sensitive *I*_sc_ over 16 days. **(D)** Time-dependent effect of IFN-γ on TEER over 16 days. **(E)** Dose-dependent effect of TNF-α on benzamil-sensitive *I*_sc_ after exposure for 7 days. **(F)** Dose-dependent effect of TNF-α on TEER after exposure for 7 days. **(G)** Time-dependent effect of TNF-α on benzamil-sensitive *I*_sc_ over 16 days. **(H)** Time-dependent effect of TNF-α on TEER over 16 days. **(I)** Dose-dependent effect of a combination of IFN-γ and TNF-α (0, 0.05, 2.5, 5, or 10 ng/mL each) on benzamil-sensitive *I*_sc_ after exposure for 7 days. **(J)** Dose-dependent effect of a combination of IFN-γ and TNF-α (0, 0.05, 2.5, 5, or 10 ng/mL each) on TEER after exposure for 7 days. Values were presented as means ± SEM (4 donors with n = 2 to 5 independent experiments per donor). Statistical significance was tested with Kruskal-Wallis, and Mann-Whitney U test was used for pairwise comparison with zero control values (* P < 0.05, ** P < 0.01, and *** P < 0.001).

Dose-dependent effect of IFN-γ and TNF-α was studied over a period of 7 days resulting in a marginal increase of benzamil-sensitive *I*_sc_ at concentrations of up to 0.05 ng/mL of IFN-γ, followed by a decrease in benzamil-sensitive *I*_sc_ at higher concentrations, which were not significantly different from control values (Fig 1A). Treatment with IFN-γ did not alter TEER at lower concentrations but caused an increase in TEER at concentrations > 0.5 ng/mL (Fig 1B). Given that ENaC activity began to decrease progressively from 0.5 ng/mL IFN-γ onwards, subsequent experiments were performed at a concentration of 1 ng/mL to ensure robust IFN-γ responses. Importantly, this concentration mirrors IFN-γ plasma levels that were observed in disease conditions associated with ENaC dysfunction, such as acute lung injury [36]. The time-dependent effect of IFN-γ on ENaC activity and barrier function was monitored over 16 days. While ENaC activity did not change throughout the period studied (Fig 1C), the presence of 1 ng/mL IFN-γ resulted in a significant increase in TEER on day 8, with a maximum increase observed on day 14 when compared to time point zero (448.1 ± 45.5, P < 0.05, and 279.3 ± 21.7 Ω·cm^-2^, P < 0.04; Fig 1D).

These findings suggest that ENaC activity and barrier function are largely unaffected by IFN-γ at lower concentrations but cause an increase in TEER with longer treatment duration and higher concentrations.

In contrast, treatment with increasing concentrations of TNF-α significantly decreased benzamil-sensitive *I*_sc_ in HBECs starting at concentrations of ≥ 0.05 ng/mL (Fig 1E) which was similar to plasma levels observed in patients with acute lung injury, severe COPD or pneumonia [37–41]. The reduction in benzamil-sensitive *I*_sc_ plateaued at around 10 ng/mL, while TEER increased when exposed to 10 and 20 ng/mL of TNF-α (319.1 ± 19.9 Ω·cm^-2^; P < 0.02, and 341.7 ± 21.2 Ω·cm^-2^; P < 0.003) when compared to 0 ng/mL (241.4 ± 14.6 Ω·cm^-2^; Fig 1F). Given the considerable reduction in benzamil-sensitive *I*_sc_ at concentrations of ≥ 0.5 ng/mL, TNF-α was used at 1 ng/mL for all subsequent experiments to ensure complete inhibition. When HBECs were incubated with 1 ng/mL TNF-α for 16 days, benzamil-sensitive *I*_sc_ progressively decreased over time. Significant decrease in benzamil-sensitive *I*_sc_ was observed after 12 days, with a maximum reduction of benzamil-sensitive *I*_sc_ on day 16 (day 12: 14.7 ± 1.8, day 14: 15.8 ± 2.2, and day 16: 14.2 ± 2.3 vs 28.2 ± 4.4 μA·cm^-2^; P < 0.03, P < 0.04, and P < 0.04, respectively; Fig 1G). Interestingly, TEER did not change when HBECs were exposed to 1 ng/mL TNF-α for 16 days (Fig 1H).

The observation that TNF-α disrupts ENaC activity considerably at concentrations seen in Th1-mediated airway inflammation [41] suggests its potential role in the pathogenesis of these conditions.

Acute Th1-mediated inflammatory responses following viral infections often involve increased production of both IFN-γ and TNF-α. When HBECs were exposed to increasing concentrations of an IFN-γ and TNF-α cocktail for 7 days, benzamil-sensitive *I*_sc_ began to decrease at concentration as low as 2.5 ng/mL, with a substantial reduction observed at 10 ng/mL compared to control cells (13.4 ± 2.9 vs 31.2 ± 2.6 μA·cm^-2^, P < 0.001; Fig 1I). However, no significant changes were observed in TEER (Fig 1J).

This suggests that IFN-γ may have prevented an early TNF-α-triggered decrease in benzamil-sensitive *I*_sc_ at lower concentrations, with the inhibitory effect tapering down at higher TNF-α concentrations, suggesting concentration-dependent regulatory interactions between the two cytokines.

### IL-13 and IL-4 cause a significant reduction in ENaC activity and barrier function

**Fig 2:**
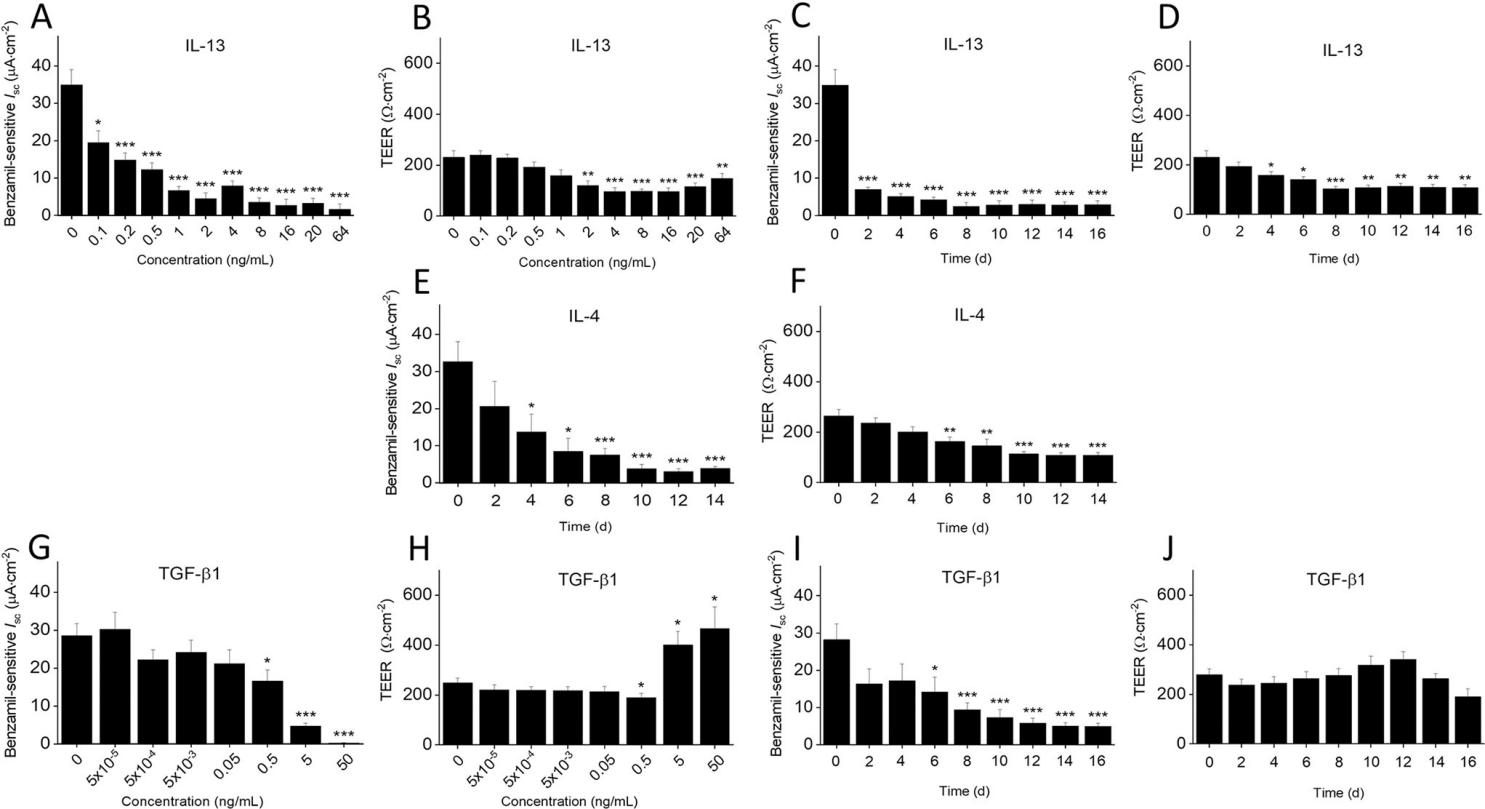
Dose- and time-dependent effect of IL-13, IL-4, and TGF-β1 on benzamil-sensitive *I*_sc_ and TEER in HBECs. Dose-dependent effect of the cytokines on benzamil-sensitive *I*_sc_ and TEER was analyzed in primary, fully differentiated HBECs that were incubated with increasing concentrations of IL-13 (0.1 to 64 ng/mL) and TGF-β1 (5 x 10^−5^ to 50 ng/mL) for 14 or 7 days, respectively. The time-dependent effect of IL-4 (2 ng/mL), IL-13 (20 ng/mL), and TGF-β1 (1 ng/mL) on benzamil-sensitive *I*_sc_ and TEER was analyzed in HBECs that were incubated for 14 to 16 days, and data were recorded on days 0, 2, 4, 6, 8, 10, 12, 14, and 16. Delta *I*_sc_ was calculated from *I*_sc_ before and 15 minutes after adding 6 μM benzamil apically to the Ussing chamber. TEER was recorded 30 minutes after mounting the cell culture inserts in Ussing chambers. **(A)** Dose-dependent effect of IL-13 on benzamil-sensitive *I*_sc_ after exposure for 14 days. **(B)** Dose-dependent effect of IL-13 on TEER after exposure for 14 days. **(C)** Time-dependent effect of IL-13 on benzamil-sensitive *I*_sc_ over 16 days. **(D)** Time-dependent effect of IL-13 on TEER over 16 days. **(E)** Time-dependent effect of IL-4 on benzamil-sensitive *I*_sc_ over 14 days. **(F)** Time-dependent effect of IL-4 on TEER over 14 days. **(G)** Dose-dependent effect of TGF-β1 on benzamil-sensitive *I*_sc_ after exposure for 7 days. **(H)** Dose-dependent effect of TGF-β1 on TEER after exposure for 7 days. **(I)** Time-dependent effect of TGF-β1 on benzamil-sensitive *I*_sc_ over 16 days. **(J)** Time-dependent effect of TGF-β1 on TEER over 16 days. Values are presented as means ± SEM (4 donors with n = 2 to 5 independent experiments per donor). Statistical significance was tested with Kruskal-Wallis, and Mann-Whitney U test was used for pairwise comparison with zero control values (* P < 0.05, ** P < 0.01, and *** P < 0.001).

When HBECs were exposed to IL-13 at increasing concentrations for a period of 14 days, a profound decrease in benzamil-sensitive *I*_sc_ was observed, starting at a concentration as low as 0.1 ng/mL, compared to control conditions (19.5 ± 2.9 vs 35.0 ± 3.9 μA·cm^-2^, P < 0.03; Fig 2A). TEER reduction occurred at 2 ng/mL of IL-13 (120.3 ± 16.3 Ω·cm^-2^, P < 0.004), with a peak decrease observed at 16 ng/mL (97.0 ± 12.9 Ω·cm^-2^, P < 0.001; Fig 2B). Incubation of HBECs with 20 ng/mL IL-13 for a period of 16 days reduced benzamil-sensitive *I*_sc_ as early as day 2 (7.0 ± 0.6 vs 35.0 ± 4.0 μA·cm^-2^, P < 0.001) with a maximum reduction on day 8 (Fig 2C). TEER decreased gradually over time and plateaued on day 8 (104.2 ± 6.8 vs 230.8 ± 24.5 Ω·cm^-2^, P < 0.001; Fig 2D). Similarly, incubation of the cells with IL-4 for 14 days at a lower concentration (2 ng/mL) caused a decrease in benzamil-sensitive *I*_sc_ starting as early as day 4 (13.7 ± 4.5 vs 32.7 ± 5.0 μA·cm^-2^, P < 0.03; Fig 2E). The maximum reduction in benzamil-sensitive *I*_sc_ was seen on day 12 (3.1 ± 0.7 μA·cm^-2^; P < 0.001) and remained low for the remainder of the study period. In the presence of IL-4, barrier function decreased as early as day 6 with maximum inhibition on day 12 (108.7 ± 9.4 vs 265.0 ± 23.0 Ω·cm^-2^, P < 0.001; Fig 2F).

The immediate and potent inhibitory effect of IL-13 and IL-4 on ENaC activity and epithelial barrier function suggests that both cytokines play a pivotal role in the early stages of inflammation, failing to clear excessive airway secretions in Th2-mediated disease conditions.

### TGF-β1 decreases ENaC activity while preserving barrier function

Incubation of HBECs with TGF-β1 for 7 days showed a concentration-dependent decrease in benzamil-sensitive *I*_sc_ starting at 0.5 ng/mL (16.6 ± 2.8 vs 28.5 ± 3.1 μA·cm^-2^, P < 0.04; Fig 2G), with further reduction of ENaC activity at 5 and 50 ng/mL (4.8 ± 0.7 and 0.3 ± 0.1 μA·cm^-2^, P < 0.001). TEER significantly decreased at 0.5 ng/mL of TGF-β1 (189.4 ± 15.4 vs 248.7 ± 18.3 Ω·cm^-2^, P < 0.03; Fig 2H), but then increased at 5 and 50 ng/mL (400.4 ± 51.6 and 466.9 ± 80.6, P < 0.02 and P < 0.03, respectively). Exposure to 1 ng/mL of TGF-β1 decreased benzamil-sensitive *I*_sc_, starting on day 6 with a maximum reduction observed on day 14 (5.0 ± 0.8 vs 28.2 ± 4.4 μA·cm^-2^, P < 0.001; Fig 2I). However, TEER remained unaffected for the duration of the study (Fig 2J).

The dose- and time-dependent inhibitory effect of TGF-β1 on ENaC activity suggests a significant role in pathological conditions associated with ENaC dysfunction.

### AA-T enhances ENaC function in Ussing chambers and patch clamp studies in the presence of high IL-13 concentrations

**Fig 3:**
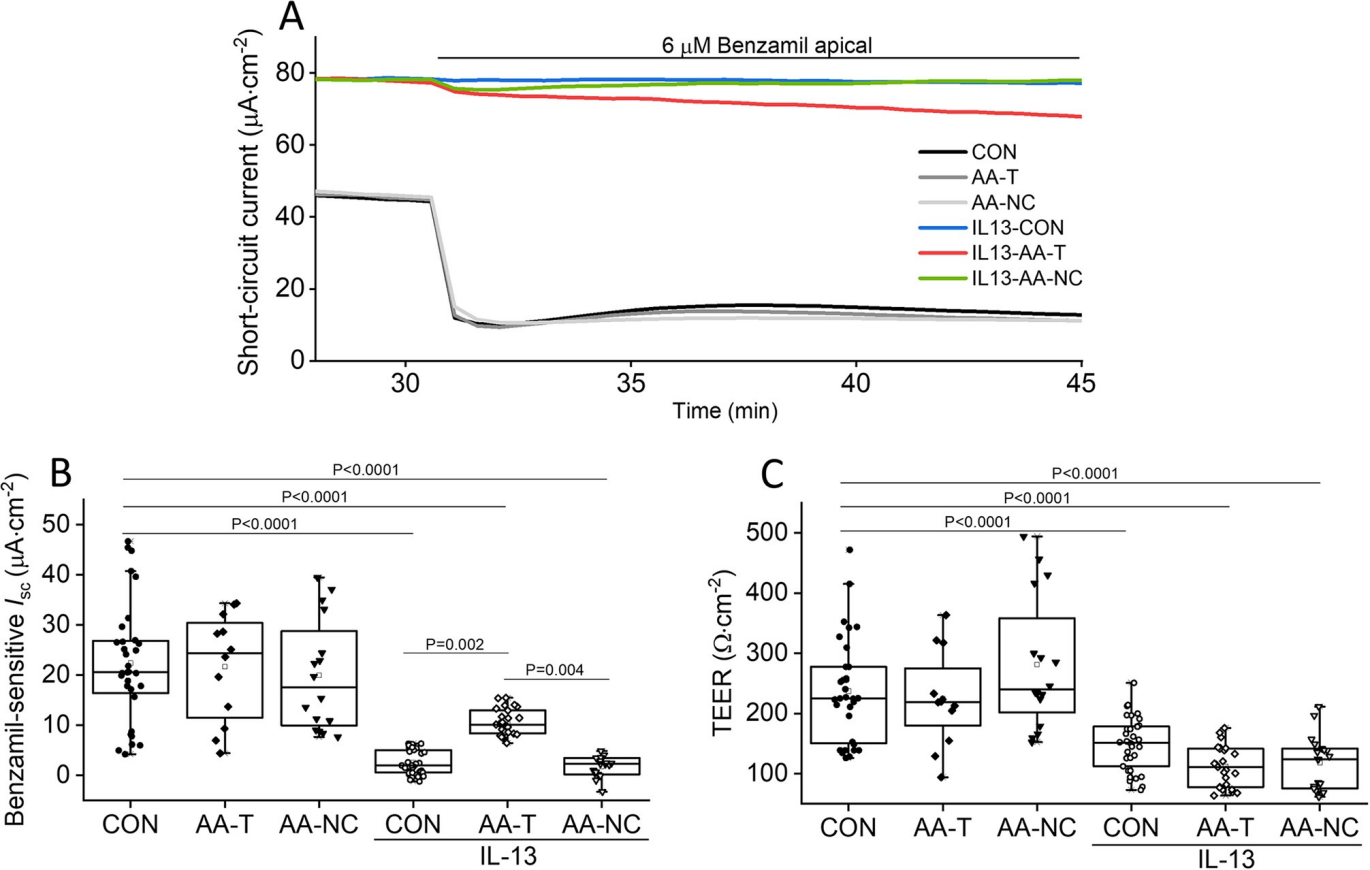
AA-T but not AA-NC increases benzamil-sensitive *I*_sc_ in IL-13-treated HBECs. The effect of AA-T and AA-NC (negative control) on benzamil-sensitive *I*_sc_ and TEER was analyzed in primary, fully differentiated HBECs that were incubated with 20 ng/mL IL-13 for 14 days. Delta *I*_sc_ was calculated from *I*_sc_ before and 15 minutes after adding 6 μM benzamil apically to the Ussing chamber. TEER was recorded 30 minutes after mounting the cell culture inserts in Ussing chambers. **(A)** Representative short-circuit current traces after exposure of normal untreated HBECs, and HBECs treated with 20 ng/mL IL-13 for 14 days to Ringer (CON), AA-T, or AA-NC in response to benzamil. **(B)** Effect of Ringer, AA-T, and AA-NC on benzamil-sensitive *I*_sc_. **(C)** Effect of Ringer, AA-T, and AA-NC on TEER. Values are presented as median (line), mean (open square), 25 and 75 percentiles (box), and outliers (whisker), (4 donors with n = 3 to 9 independent experiments per donor). Statistical significance was tested with one-way ANOVA, and Bonferroni test was used for pairwise comparisons (P < 0.05).

Previous research has demonstrated that specific amino acids can increase the expression and function of membrane transporters, channels, and tight junction proteins, thereby justifying the use of amino acids to enhance ENaC activity [32–34]. A formulation comprising five amino acids (AA-T) that showed promise in preliminary experiments for their potential to improve ENaC expression and function was therefore tested for their ability to improve ENaC expression and function in HBECs incubated with 20 ng/mL of IL-13 for 14 days. Representative traces of short-circuit current changes in normal and IL-13-treated HBECs in response to Ringer, AA-T, or AA-NC are presented in Fig 3A. When normal HBECs were bathed in the amino acids (AA-T and AA-NC) in Ussing chambers, benzamil-sensitive *I*_sc_ did not change compared to Ringer control (21.7 ± 3.1 μA·cm^-2^ vs 19.9 ± 2.8 μA·cm^-2^ vs 22.4 ± 2.1 μA·cm^-2^; Fig 3B). The same response pattern applied to TEER (Fig 3C). When HBECs were challenged with IL-13 for 14 days, the substantial decrease in benzamil-sensitive *I*_sc_ ameliorated significantly in response to AA-T (2.5 ± 0.4 μA·cm^-2^ vs 10.6 ± 0.5 μA·cm^-2^, P = 0.002), but the values remained lower than those of control cells (P < 0.001; Fig 3B). Conversely, IL-13-treated cells exposed to a set of amino acids selected to decrease ENaC activity (AA-NC) exhibited persistently lower benzamil-sensitive *I*_sc_ (1.8 ± 0.5 μA·cm^-2^), with values similar to IL-13-treated cells exposed to Ringer. ENaC function showed improvement within 30 minutes after exposure to AA-T, but full restoration did not occur during the study period. In addition, IL-13-induced barrier disruption remained unchanged by AA-T (Fig 3C). This suggests that further optimization is needed in terms of incubation time and amino acid concentrations.

**Fig 4:**
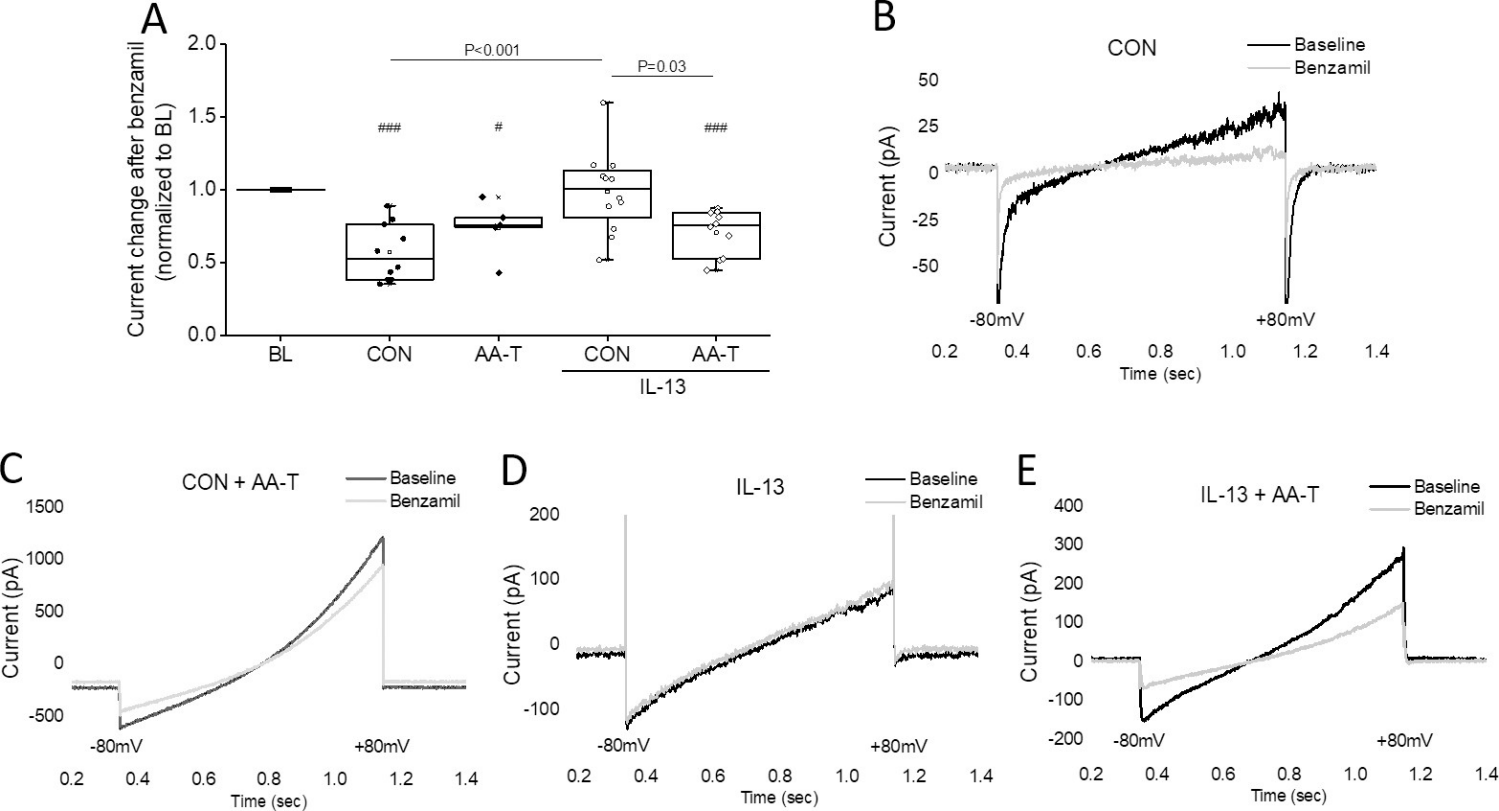
AA-T enhances sodium current in whole-cell recordings in IL-13-treated HBECs. **(A)** Changes of sodium current (fold change) in response to 6 μM benzamil (at -80 mV) in untreated HBECs, and HBECs treated with 20 ng/mL of IL-13 for 4 days when exposed to AA-T, or Ringer (CON). Values from one donor (n = 5 to 12 independent experiments = single cells) are presented as median (line), mean (open square), 25 and 75 percentiles (box), and outliers (whisker). Individual values were normalized to the corresponding baseline values (BL). Statistical significance was tested with a paired t-test for comparison of the values before and after treatment with benzamil (^#^ P < 0.05, and ^###^ P < 0.001). One-way ANOVA followed by the Bonferroni test was used for pairwise comparisons between the treatment groups (P < 0.05). **(B-E)** Representative traces of whole-cell recordings using a ramp protocol from -80 mV to +80 mV showing the effect of benzamil on ENaC-mediated sodium current in untreated HBECs, and HBECs treated with 20 ng/mL of IL-13 for 4 days. **(B)** Untreated HBECs exposed to Ringer (CON), **(C)** Untreated HBECs exposed to AA-T, **(D)** IL-13-treated HBECs exposed to Ringer (CON), and **(E)** IL-13-treated HBECs exposed to AA-T.

Long-term treatment of HBECs with IL-13 alters the expression of ion channels and also cause morphological changes that influence ENaC function either directly or indirectly. Thus, patch clamp whole-cell recordings on benzamil-sensitive current were performed in HBECs exposed to IL-13 for 4 days (Fig 4A–4E). These recordings supported the findings on ENaC function as demonstrated in Ussing chamber studies in the presence of AA-T. Because the patch clamp experiments were designed to mimic the Ussing chamber experimental conditions, the currents were measured 15 minutes after addition of 6 μM benzamil. Treatment with benzamil caused a significant decrease of the sodium current in normal untreated cells exposed to both, Ringer and AA-T (0.57 ± 0.06 and 0.74 ± 0.09 fold change normalized to baseline values, P < 0.001 and P < 0.04; Fig 4A–4C). Cells treated with 20 ng/mL of IL-13 for 4 days and bathed in Ringer solution (CON) did not respond to benzamil, and the sodium current remained unchanged (0.99 ± 0.08 fold; Fig 4D). The changes in ENaC current observed in patch clamp studies could be a result of alterations in the expression levels of the channel.

However, when IL-13-challenged HBECs were exposed to AA-T for 15 minutes, the magnitude of benzamil-sensitive sodium current increased significantly when compared to IL-13-challenged HBECs exposed to Ringer solution for 15 minutes (0.71 ± 0.05 fold change, P < 0.001; Fig 4A and 4E). The representative data shown in Fig 4B–4E are from a ramp protocol from -80 mV to +80 mV applied to the single cell every 90 sec for a maximum of 15 minutes in normal and IL-13-treated HBECs before and after blocking ENaC activity with benzamil in the presence of Ringer (CON) or AA-T. The current under basal conditions showed an initially inward current followed by an outward current towards more positive potentials.

The increase in ENaC activity observed in IL-13-challenged HBECs with AA-T suggests that AA-T could be a potential therapeutic agent to correct ENaC dysfunction in Th2-mediated inflammatory conditions. However, further optimization is required to determine the amino acid concentration, duration, timing, and mode of delivery.

### AA-T enhances ENaC function by increasing membrane proteins and gene expression

**Fig 5:**
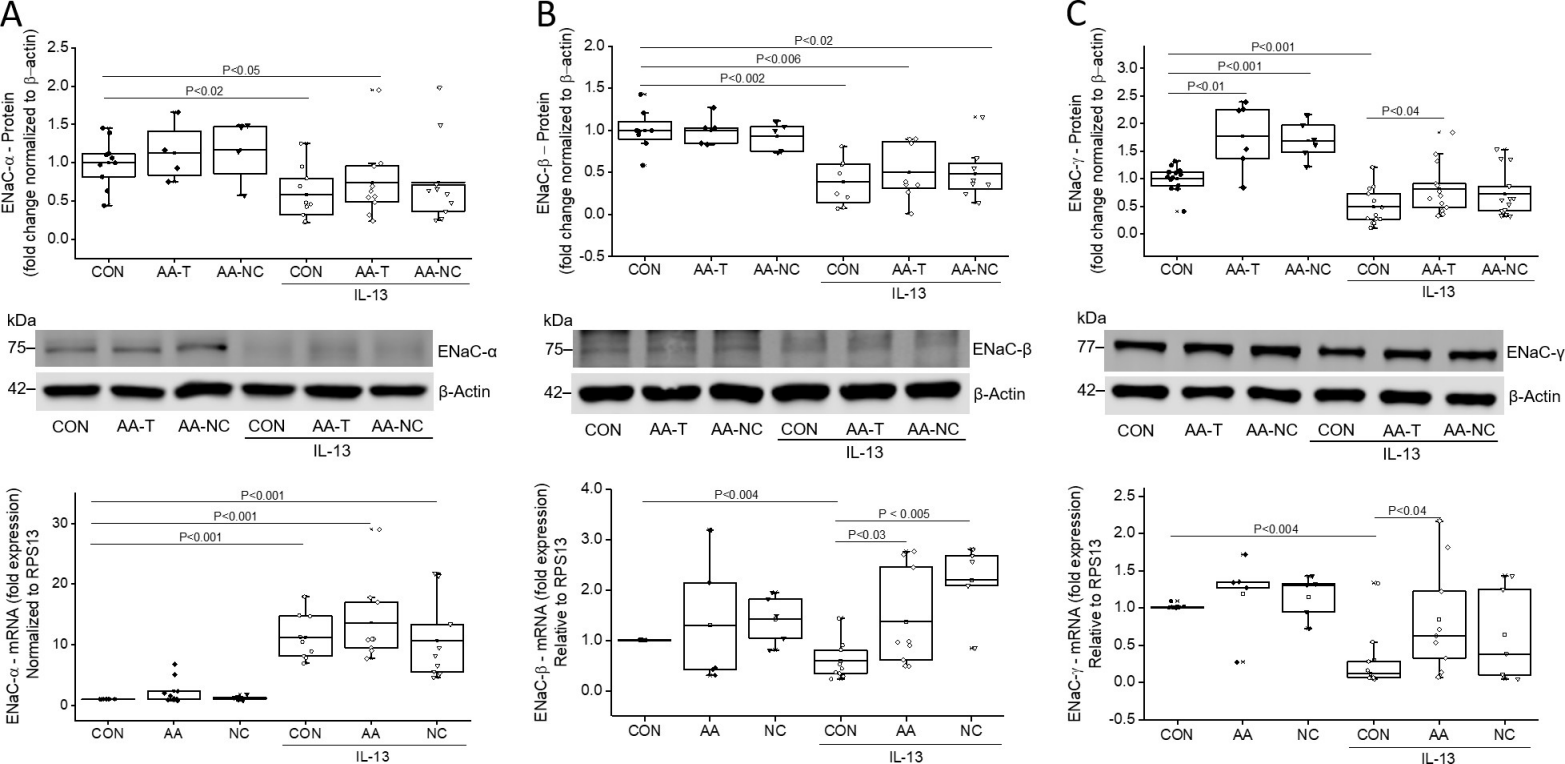
AA-T alters apical membrane protein, and mRNA expression levels in untreated, and IL-13-treated HBECs. Relative abundance of α-ENaC (~75 kDa), β-ENaC (~75 kDa), and γ-ENaC protein (~77 kDa) within the total membrane fraction are shown for untreated HBECS, or HBECs treated with 20 ng/mL of IL-13 for 10 days followed by exposure to Ringer (CON), AA-T, or AA-NC for 1 hour (upper panels). Values from 2 donors (n = 2 to 11 independent experiments per donor) were calculated as fold change with respect to β-actin (~42 kDa). Representative western blots for α-, β-, and γ-ENaC protein expression are shown in the middle panels. mRNA expression levels of α-, β-, and γ-ENaC were analyzed by real-time qPCR (lower panels). HBECs were treated with IL-13 for 7 days followed by exposure to Ringer (CON), AA-T, or AA-NC for 5 days. Values were calculated as fold change with respect to RPS13 using the Pfaffl method [35]. Values from two donors with n = 4 to 13 independent experiments per group were presented as median (line), mean (open square), 25 and 75 percentiles (box), and outliers (whisker). Statistical significance was tested with Kruskal-Wallis test, and the Mann-Whitney U test was used for pairwise comparisons (P < 0.05). **(A)** α-ENaC membrane protein expression (upper panel), representative western blot (middle panel), and mRNA levels (lower panel), **(B)** β-ENaC membrane protein expression (upper panel), representative western blot (middle panel), and mRNA levels (lower panel), **(C)** γ-ENaC membrane protein expression (upper panel), representative western blot (middle panel), and mRNA levels (lower panel).

Western blot analysis for α-ENaC, β-ENaC, and γ-ENaC protein expression in membrane fractions of HBECs exposed to amino acid formulations (AA-T and AA-NC) showed no changes in α-ENaC and β-ENaC protein levels. However, there was an increase in γ-ENaC protein levels when compared to Ringer control. In IL-13 exposed cells, the protein expression of all three ENaC subunits was significantly reduced when compared to Ringer control (Fig 5A–5C, upper panel) with relative expression levels of α-ENaC: 0.58 ± 0.11 vs 1.0 ± 0.10 fold (P < 0.02), β-ENaC: 0.39 ± 0. 10 vs 1.0 ± 0.09 fold (P < 0.002), and γ-ENaC: 0.49 ± 0.09 vs 1.0 ± 0.06 fold (P < 0.001). Thus, decreased ENaC function observed in the presence of IL-13 in the Ussing chamber and patch clamp studies correlated well with the western blot results. Despite these changes in protein levels, the gene expression of α-, β-, or γ-ENaC remained unchanged when HBECs were exposed to the amino acid formulations when compared to Ringer control (Fig 5A–5C, lower panel).

Exposure to AA-T for 1 hour did not affect membrane protein levels of α-ENaC and β-ENaC in IL-13-challenged HBECs (Fig 5A and 5B) but increased γ-ENaC protein levels to 0.82 ± 0.013 fold (P < 0.04; Fig 5C). However, γ-ENaC membrane protein did not fully recover to control levels. When HBECs were treated with IL-13 for 7 days, relative gene expression of ENaC β- and γ-subunits decreased to 0.60 ± 0.13 and 0.30 ± 0.14 fold (P < 0.004; Fig 5B and 5C), and α-ENaC mRNA increased to 11.2 ± 1.3 fold (P < 0.001; Fig 5A). After exposure of IL-13-treated cells to AA-T for 5 days, α-ENaC mRNA levels increased further to 13.6 ± 2.2 fold (P < 0.001; Fig 5A), while β-ENaC and γ-ENaC mRNA recovered to control levels (1.4 ± 0.3 and 0.8 ± 0. 2 fold P < 0.03 and P < 0.04; Fig 5A–5C). Interestingly, exposure of IL-13-treated HBECs to AA-NC also resulted in increased ENaC mRNA expression levels however, these changes did not translate into functional responses.

**Fig 6:**
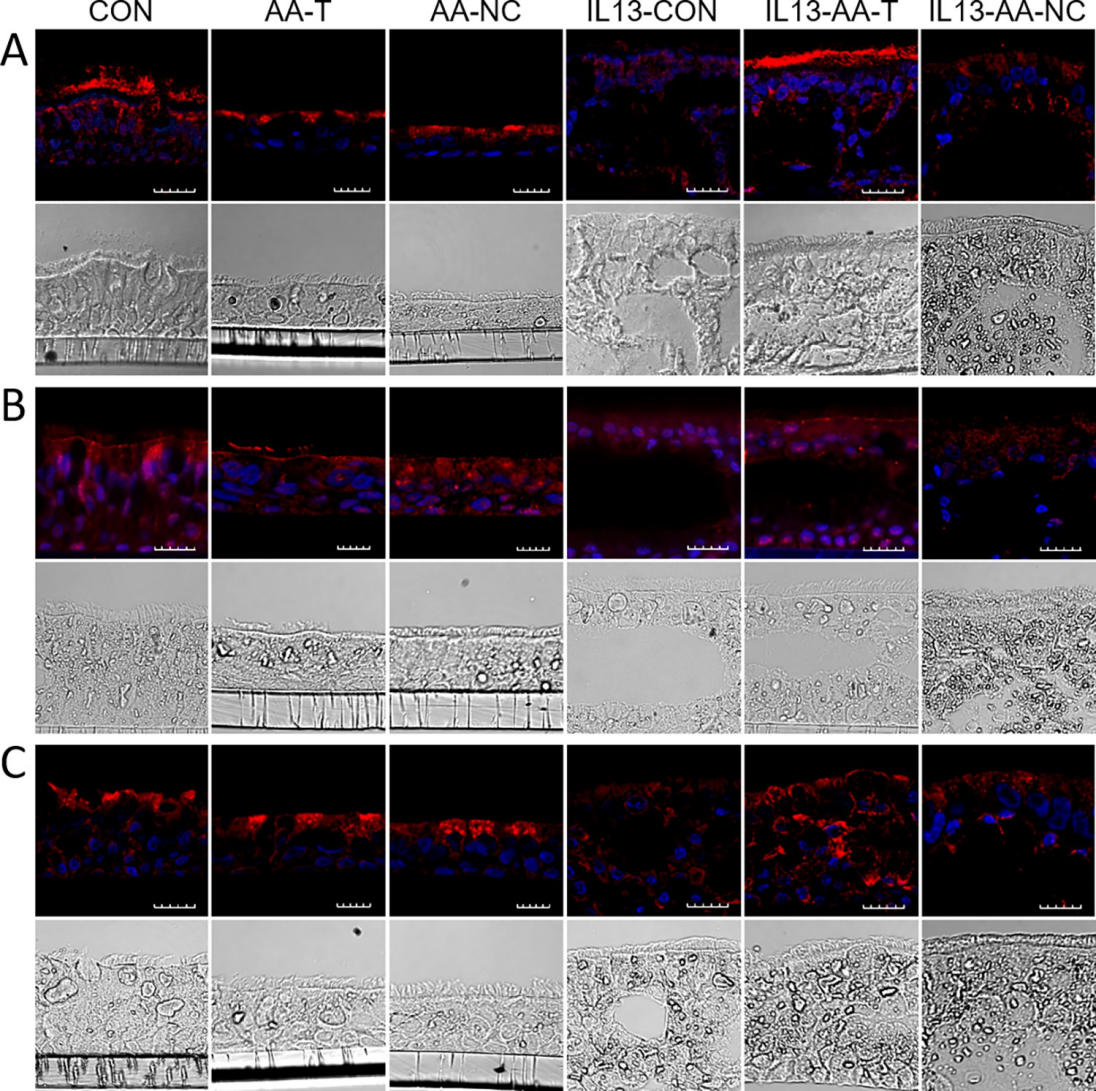
AA-T increases immunofluorescence signals of α-, β-, and γ-ENaC at the apical membrane in IL-13-treated HBECs. Expression pattern of **(A)** ENaC subunit α, **(B)** subunit β, and **(C)** subunit γ is demonstrated in untreated HBECs, and HBECs treated with 20 ng/mL of IL-13 for 14 days followed by exposure to Ringer (CON), AA-T (AA-T), or AA-NC (AA-NC) for 1 hour. ENaC expression is shown as a red signal (Alexa Fluor 647), and DAPI was used for nuclei (blue signal). The scale bar corresponds to 20 μm. All experiments were performed on 4 donors in 3 different sections.

Immunofluorescence imaging of the ENaC protein subunits showed that α-ENaC (Fig 6A), ß-ENaC (Fig 6B), and γ-ENaC (Fig 6C) expression were present along the periciliary and apical membrane at variable density, with the highest expression signal observed in ENaC α- and γ-subunits. When normal HBECs were exposed to the amino acids (AA-T or AA-NC) the immunofluorescence signal of α-ENaC and γ-ENaC appeared slightly increased. Treatment with 20 ng/mL of IL-13 for 14 days caused changes in the bronchial epithelial morphology characterized by goblet cell hyperplasia and displacement of ciliated and non-ciliated epithelial cells. The rapid increase in the goblet cell population associated with excessive mucus production resulted in the formation of goblet cell pockets within the epithelial cell layer. At the apical surface of the HBEC culture, α-ENaC and γ-ENaC seemed translocated from the apical membrane to sub-apical compartments and the cytoplasm in ciliated and non-ciliated cells, with slightly reduced, or unchanged expression patterns of ß-ENaC in the presence of IL-13. Apical exposure to AA-T for 1 hour increased the immunofluorescence signal of all three ENaC subunits along the apical and periciliary membranes in IL-13-treated cells without affecting the main morphological changes while ENaC expression remained unchanged in IL-13-treated HBECs in the presence of AA-NC.

These results demonstrate that AA-T improved ENaC function by restoring ENaC protein expression at the apical membrane. This was achieved by protein trafficking from the cytoplasm to the apical membrane, and by increasing ENaC gene expression. Increased recycling of ENaC protein or reduced endocytosis could also lead to increased ENaC protein levels at the cell surface. Further studies are however warranted to specify the identity of α-ENaC, β-ENaC, and γ-ENaC to overcome the limitations of antibody specificity and explore additional mechanisms by which the amino acids regulate ENaC expression and function.

## Discussion

The ASL comprising both a luminal mucus layer and a periciliary layer is a prerequisite for normal lung function and optimal immune response [3, 42], and ENaC plays a pivotal role in maintaining ASL homeostasis. The composition and volume of ASL are critical, and primarily regulated by active sodium transport via ENaC, followed by potential-driven anion movement through cystic fibrosis transmembrane conductance regulator, and aquaporin-mediated or paracellular transport of water [4].

Reduced ENaC activity, often due to pathological conditions, leads to excessive secretion, and accumulation of mucus and fluid in pulmonary airways, and potential lung injury [4]. Conversely, increased ENaC activity causes ASL dehydration and airway obstruction [2]. Both conditions are often triggered by cytokine production in response to sepsis, infections, or allergic reactions [4, 43]. For instance, in patients with pollen allergy, which is associated with the overexpression of the Th2 cytokines IL-4, IL-5, and IL-13, the amiloride-sensitive nasal potential difference decreases, leading to increased epithelial surface fluid volume in the upper respiratory tract with excessive rhinorrhea [44]. In early phases of acute lung injury, a common risk factor of sepsis and lung infections, excessive airway fluid contains high concentrations of pro-inflammatory Th1 cytokines like TNF-α, TGF-β1, and IFN-γ, that are known to trigger ENaC dysfunction [45, 46].

In this study, both Th1 and Th2 cytokines down-regulated ENaC expression and function in HBECs in a concentration- and time-dependent manner. Among the cytokines studied, the Th2 cytokines IL-4 and -13 had the most profound negative effect on ENaC activity and epithelial barrier function, whereas IFN-γ, TNF-α and TGF-β1 reduced ENaC activity but left TEER largely unaffected. IL-4 and IL-13 have been detected at high concentrations in patients with virus-associated pulmonary edema [47], further supporting their role in conditions with pulmonary hypersecretion.

Treatment of airway hypersecretion is primarily supportive, with few effective therapeutic agents targeting ENaC/barrier function. Exposure to the proprietary blend of five select amino acids (AA-T) partially recovered lost ENaC function in IL-13-challenged HBECs, suggesting a new approach to control fluid secretion in Th2-mediated airway dysfunction. ENaC, comprising three homologous subunits (α, β, and γ), that are independently regulated and encoded by separate genes presents a challenge for therapeutic development [48–50]. A fourth ENaC subunit, δ, is found in humans but not in rodents, contributing to around 50% of amiloride-sensitive ENaC activity across human nasal epithelial cells [2, 51, 52]. These ENaC subunits are assembled in the endoplasmic reticulum and trafficked to the apical membrane, where they form a sodium channel. The α subunit is necessary for assembling functional ENaC on the apical membrane [4]. While α and δ subunits can generate low levels of functional ENaC channels, β or γ subunits are accessory subunits and do not represent functional channels themselves [53, 54]. Although gene knockout studies demonstrated that all three subunits are essential for survival, α-ENaC is crucial for fluid clearance in neonatal and adult lungs. The relative abundance of mRNA of the three subunits in bronchial epithelial cells varies significantly, with the α subunit being the most abundant followed by subunits β and γ [54]. The β and γ subunits are more susceptible to modulation by external factors such as cytokines, as evident in the real-time qPCR and western blot data showing decreased mRNA and protein levels in the presence of IL-13. Similarly, studies have demonstrated that exposure of HBECs to 10 ng/mL IL-4 for 48 hours down-regulated mRNA expression of γ-ENaC, and to a lesser extent β-ENaC. Thus, both cytokines have a similar inhibitory effect on ENaC function possibly by sharing the same receptor and signaling cascade [23, 55]. Exposure of IL-13-treated cells to AA-T increased γ-ENaC mRNA and protein expression, suggesting that up-regulation of γ-ENaC is essential for recovery of ENaC function in our experimental setup. The considerable increase of α-ENaC mRNA, the only subunit that can form an active channel in the presence of IL-13 and AA-T suggests a compensatory mechanism by AA-T to overcome ENaC functional deficits. Because the membrane protein levels of α-ENaC and β-ENaC were not fully restored within one hour of exposure to AA-T, long-term AA-T treatment may be needed for full channel function recovery.

ENaC dysfunction and impaired airway liquid clearance are linked to lung infections. Th1 cytokines like IL-1β, IFN-γ, and TNF-α significantly contribute to bacteria- or virus-mediated acute ENaC dysfunction [46, 56–58]. Effects of IL-1β and IFN-γ on ENaC function are regulated in a p38-MAP kinase- or PKC-dependent manner, while TNF-α-mediated modulation of ENaC activity is more complex due to its pleiotropic properties. TNF-α has been shown to increase sodium currents independent of catecholamines [59–61], which is spatially distinct from the TNF-receptor binding site, responsible for downregulating both, ENaC activity and expression. In addition, TNF-α impairs lung barrier function by decreasing the expression of tight junction proteins [31, 62]. We have demonstrated that the effect of TNF-α on ENaC activity is concentration-dependent, with concentrations as low as 0.05 ng/mL significantly downregulating ENaC function. Interestingly, the epithelial barrier remained largely unaltered by TNF-α in our model. These opposing responses could be caused by the dichotomous role of TNF-α in airway liquid reabsorption, with its lectin-like activity increasing clearance and its receptor-dependent activity decreasing ENaC function [62–64]. Variations in TNF receptor expression levels, distribution, concentration, and/or exposure time could contribute to these conflicting responses.

TNF-α, an early and most potent pro-inflammatory cytokines, often correlates with disease severity and usually works with IFN-γ, which further triggers TNF-α production via activation of NF-kB, exacerbating airway secretion [46, 57, 65, 66]. As a member of the type II IFN family, IFN-γ has potent antiviral activity by promoting antigen presentation, stimulating cytotoxic T-cells, CD4+ and CD8+ memory cells, and activating natural killer cell function [67, 68]. Early and sustained low-level IFN-γ release could exert a protective effect by enhancing barrier function, as demonstrated in the present study. Interestingly, IFN-γ increased paracellular permeability in endothelial and epithelial monolayers [69], but had anti-inflammatory properties and improved epithelial barrier function in airway epithelial cells, indicating its pleiotropic properties [70, 71]. The exact mechanism by which IFN-γ exerts its effect on TEER is not known, but IFN-γ can modulate the expression of distinct tight junction proteins and reorganize cytoskeletal components [18, 69, 72]. The complex interplay between IFN-γ and TNF-α was evident when a combination of IFN-γ and TNF-α inhibited ENaC function in HBECs at concentrations > 2.5 ng/mL while at lower concentrations, the protective properties of IFN-γ compensated the inhibitory effect of TNF-α. This effect was reversed at higher concentrations of TNF-α, suggesting that the downregulation of ENaC function by Th1 cytokines depends on the concentration of TNF-α and thus, on the severity of the inflammatory response [56, 73]. In contrast, the combination of IFN-γ and TNF-α had no effect on barrier function at the concentrations used. In studies on human airway cells, TEER decreased by 92% after exposure to a combination of 100 ng/mL IFN-γ and 10 ng/mL TNF-α for 3 days [18], concentrations that exceed the circulating amounts of IFN-γ and TNF-α observed in patients with severe COVID [74–76], suggesting that IFN-γ and TNF-α act in a time- and concentration-dependent manner.

In addition, there is a positive association between TNF-α and TGF-β, with the former representing the acute pro-inflammatory route and the latter the classic immunosuppressive pathway [77]. However, the role of TGF-β is more complex, exhibiting pro-inflammatory properties that vary depending on the microenvironment and type of inflammation. TGF-β plays a pivotal role in maintaining peripheral immune tolerance and homeostasis by suppressing Th1 and Th2 cell differentiation, natural killer cell development and function, B cell survival, and immunoglobulin synthesis while promoting the development of regulatory T cells, and regulating the tolerogenic and pathogenic functions of dendritic cells and macrophages [78–80]. TGF-β consists of three isoforms (TGF-β1, 2, and 3), and is the most abundant cytokine in most tissues, including the lung. It is generally involved in growth, proliferation, and differentiation, but it also contributes to the anti-inflammatory Treg immune response that inhibits the activation of pro-inflammatory cytokines such as IFN-γ, TNF-α, and interleukins [81, 82]. Despite its immuno-suppressive nature, TGF-β is a potent chemoattractant and initiates inflammation together with IL-6 and via Th17 differentiation of naïve cells [83]. Studies have also shown that TGF-β is implicated in transepithelial ion transport and down-regulates gene and protein expression of ENaC-α resulting in reduced amiloride-sensitive Na uptake and fluid transport [27, 29].

Downregulation of ENaC activity by TGF-β1 started at very low concentrations, and as early as 2 days after exposure when tested independently of other cytokines, with no inhibitory effect on barrier function. These early effects of TGF-β1 could be triggered by the rapid production of reactive oxygen species, which drives the internalization of β-ENaC, the subunit responsible for cell-surface stability [29].

## Conclusions

**Fig 7:**
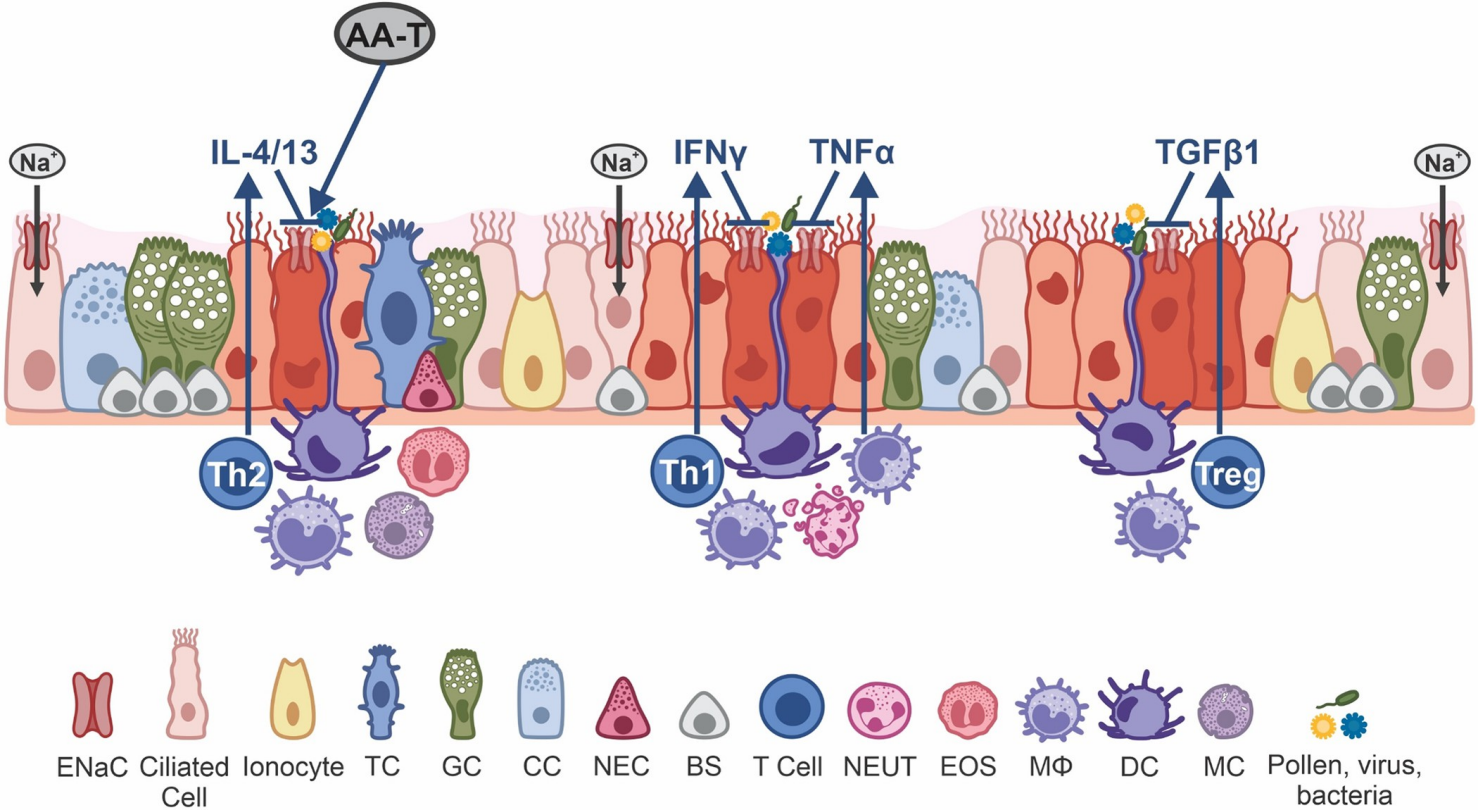
Schematic illustration of the effect of cytokines and AA-T on ENaC activity in bronchial epithelial cells. Th1-mediated cytokines, such as IFNγ and TNFα, Th2 cytokines like IL-4 and IL-13, and Treg-mediated response involving TGFβ1 can inhibit ENaC function in bronchial epithelial cells. Notably, Th1 cytokines appear to have the most significant impact on ENaC, affecting apical sodium and fluid absorption in HBECs, and the compromised ENaC activity can be restored by a proprietary blend of specific amino acids (AA-T). (↑ increase; ⊥ inhibition; BS–basal cell, CC–club cell, DC–dendritic cell, ENaC–Epithelial Sodium Channel, EOS–eosinophil, GC–goblet cell, MC–mast cell, MΦ–macrophage, NEC–neuroendocrine cell, NEUT–neutrophil, TC–tuft cell). The illustration was created using Corel Draw, version 21.3.0.755 (2019).

This study shows that pro- and anti-inflammatory cytokines can inhibit ENaC activity in HBECs in a time- and concentration-dependent manner, with Th2 cytokines having the most profound impact on ENaC dysfunction (Fig 7). However, changes in barrier function were less pronounced. A proprietary blend of amino acids (AA-T) was able to partially recover ENaC activity in IL-13-challenged cells at both the transcriptional and translational levels, counteracting the adverse effects of IL-13. Further studies will explore the mechanisms of these amino acids, and their relationship with ENaC transcription, translation, trafficking, and function. With no approved drugs available to increase ENaC function, AA-T could be considered a potential therapeutic agent for treating pulmonary hypersecretion in affected patients as a standalone, or complementary agent in combination with other treatment options. AA-T is characterized by its safety profile, simplicity, and intrinsic appeal which could maximize the effect of standard therapy while minimizing complications in affected patients.

## Supporting information

S1 Raw images (PDF)
