# Supplementary material for: Select amino acids recover cytokine-altered ENaC function in human bronchial epithelial cells
Source: PLoS One. 2024 Jul 25;19(7):e0307809. doi: 10.1371/journal.pone.0307809 (PMC11271875; doi:10.1371/journal.pone.0307809)

ENaC-α Western blot representative image

ENaC-α imaged using IRDye 800CW secondary antibody

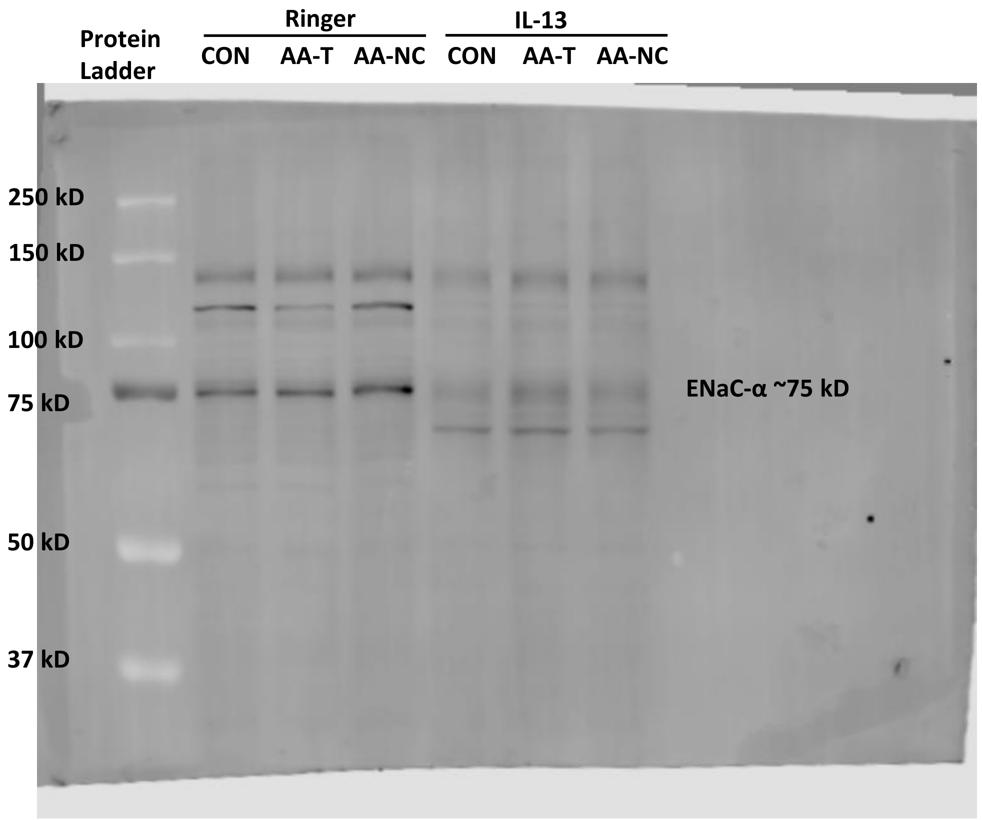

β-actin imaged using IRDye 680CW secondary antibody
(same membrane as that of ENaC-α)

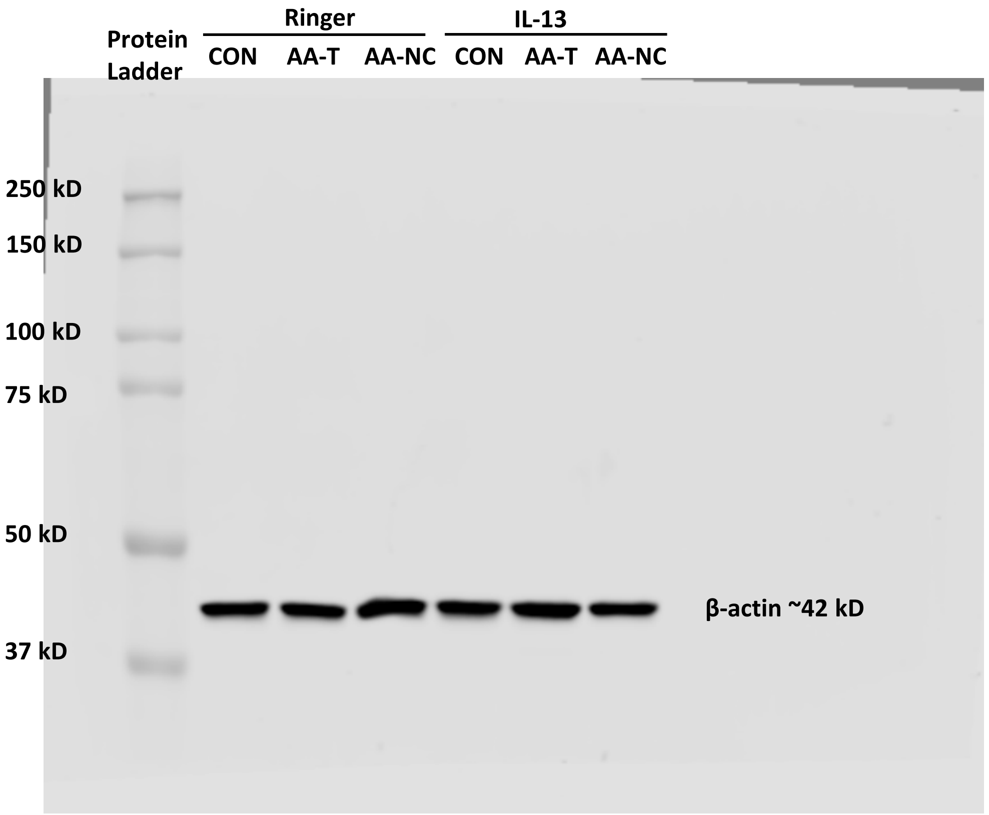

ENaC-β Western blot representative image

ENaC-β imaged using IRDye 800CW secondary antibody

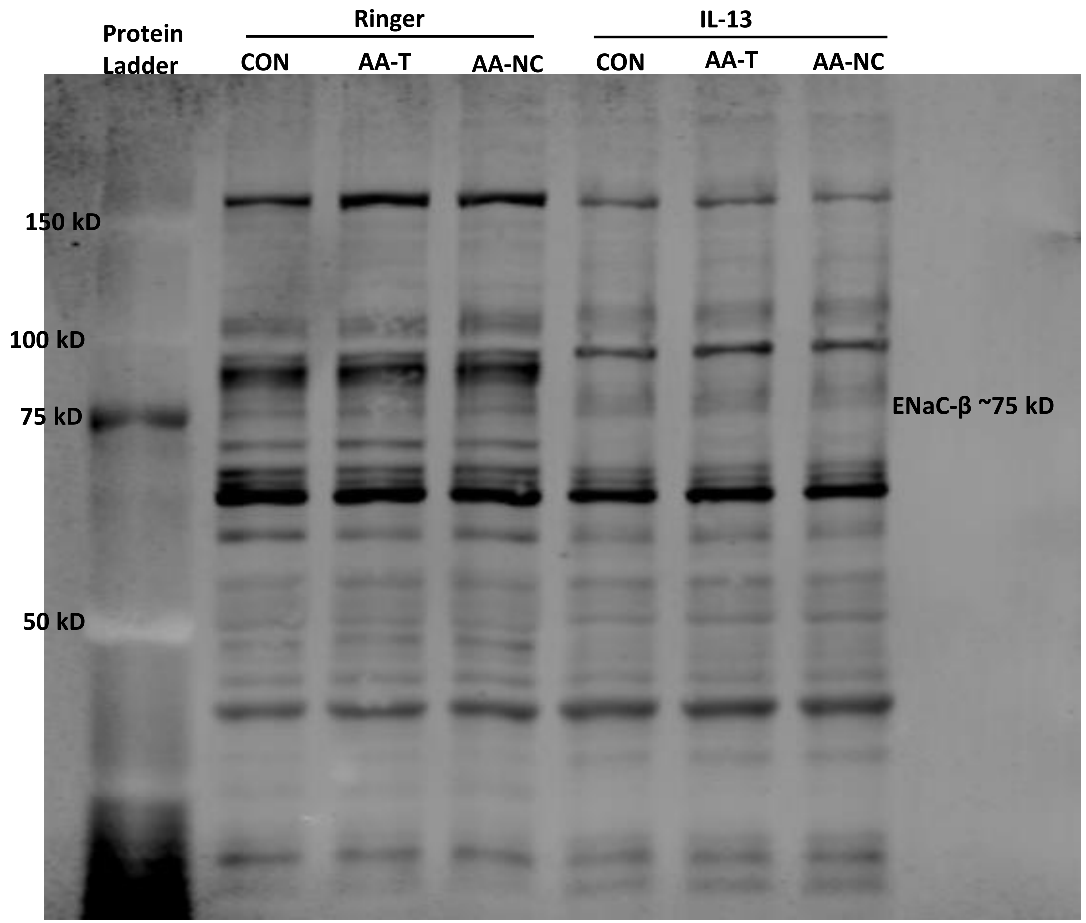

β-actin imaged using IRDye 680CW secondary antibody
(same membrane as that of ENaC-β)

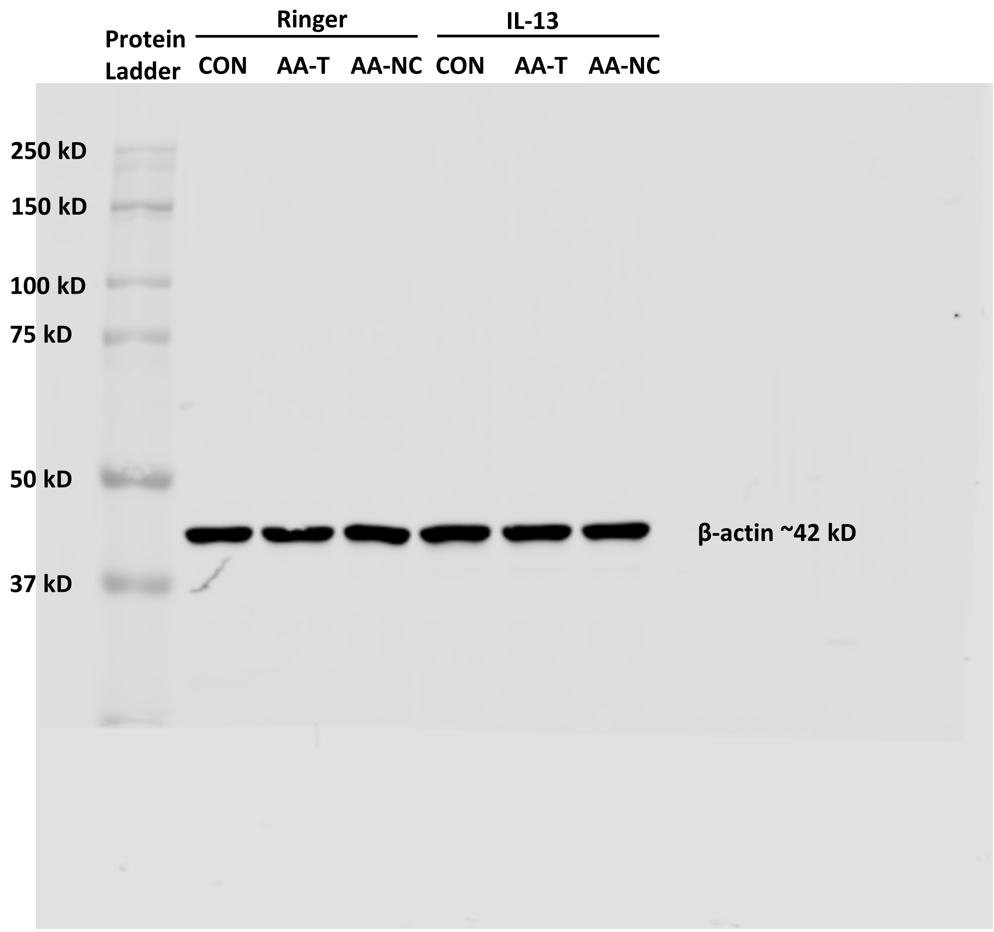

ENaC-γ Western blot representative image

ENaC-γ imaged using IRDye 800CW secondary antibody

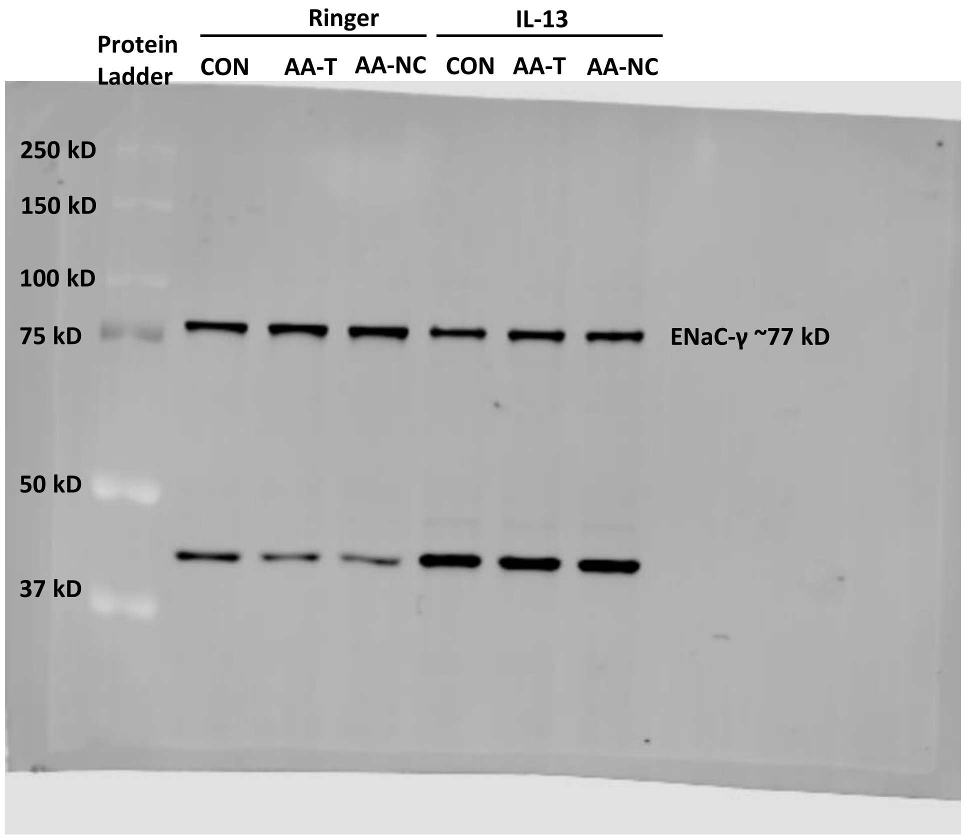

β-actin imaged using IRDye 680CW secondary antibody
(same membrane as that of ENaC-γ)

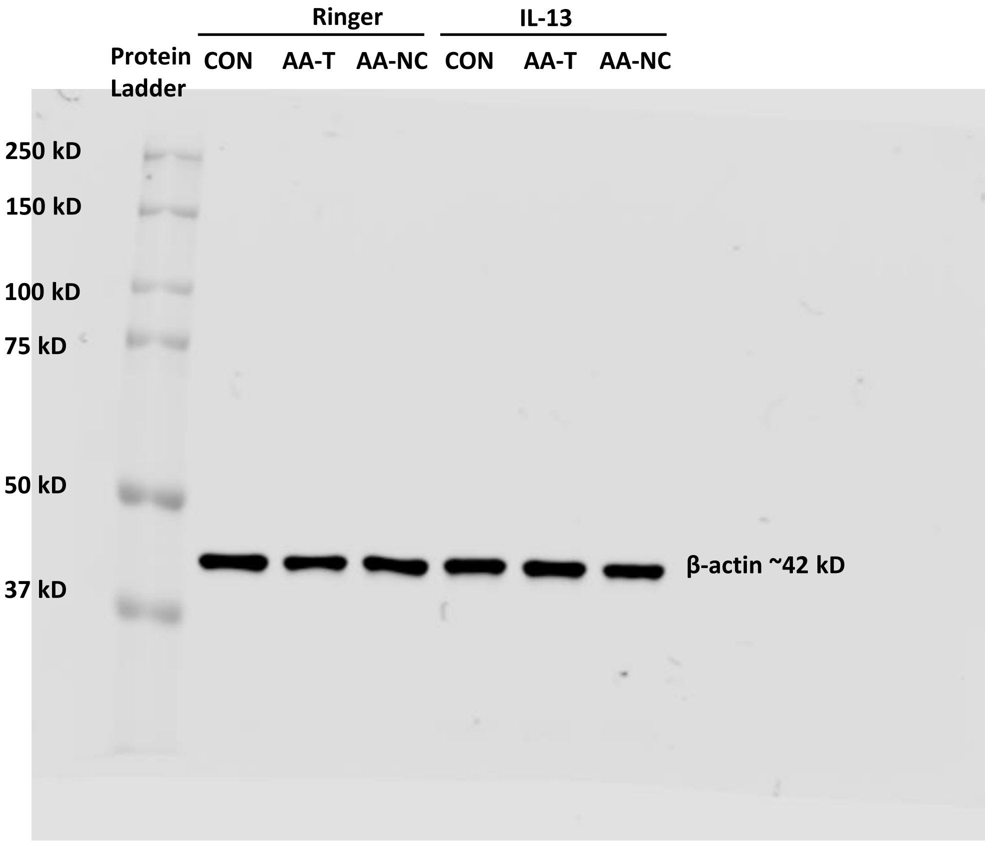

ENaC-α Western blot images

ENaC-α Blot 1

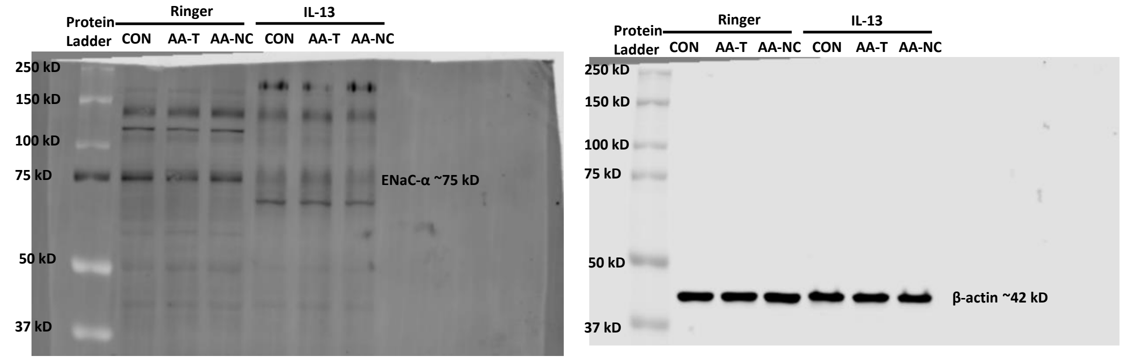

ENaC-α Blot 2

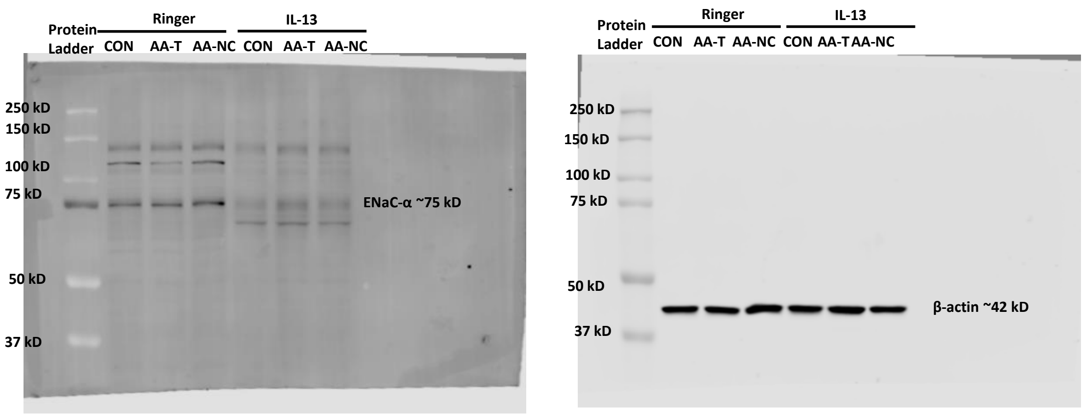

ENaC-α Blot 3

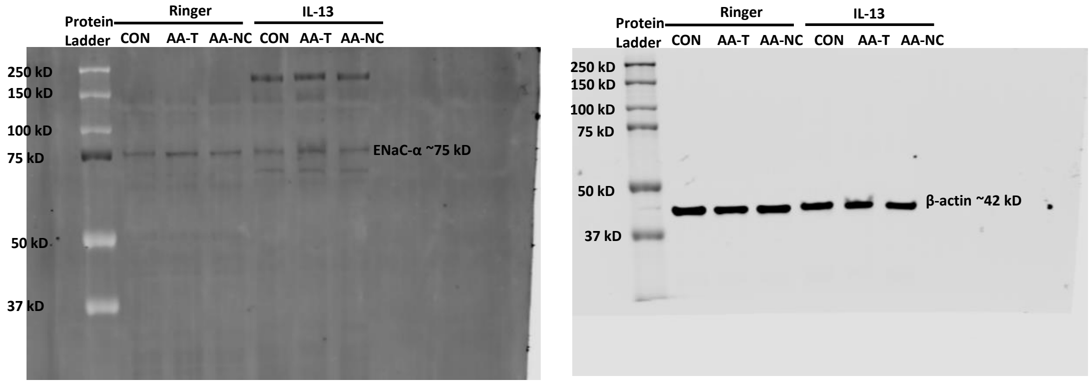

ENaC-α Blot 4

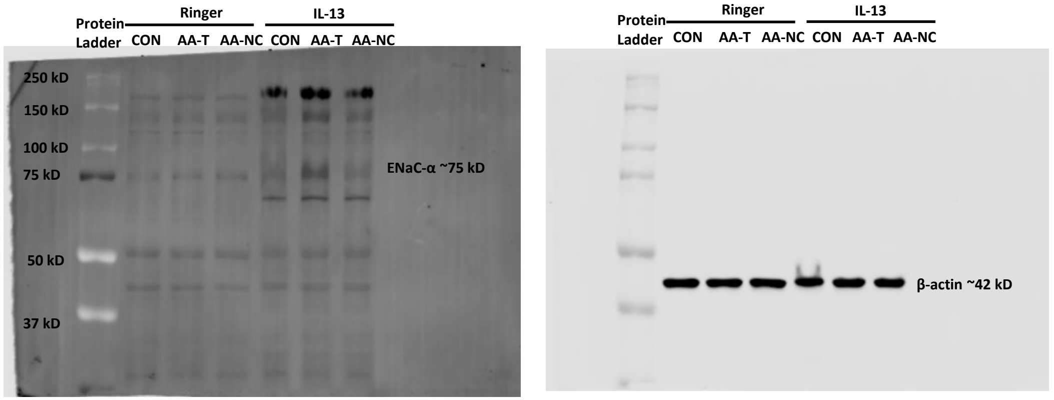

ENaC-α Western blot images

ENaC-α Blot 5

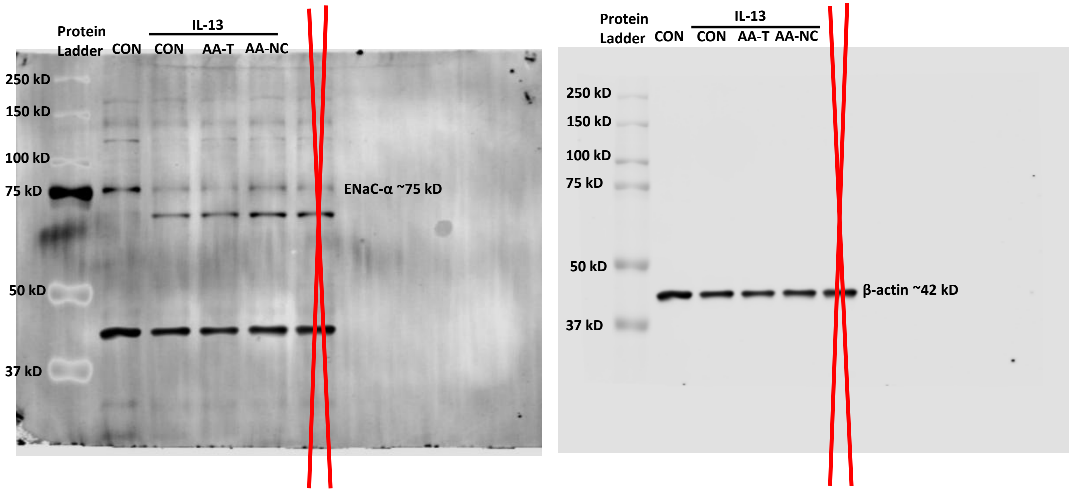

ENaC-α Blot 6 & 7

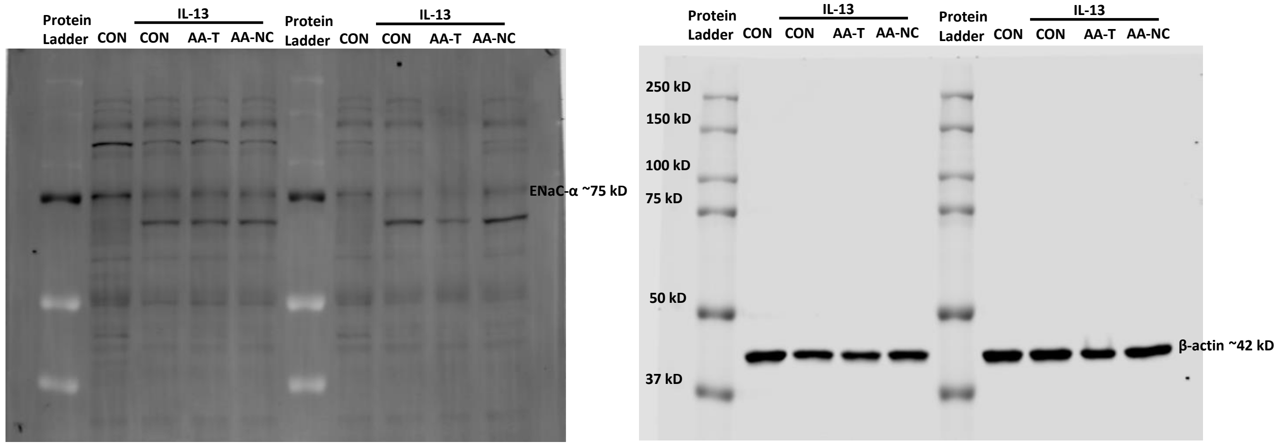

ENaC-α Blot 8 & 9

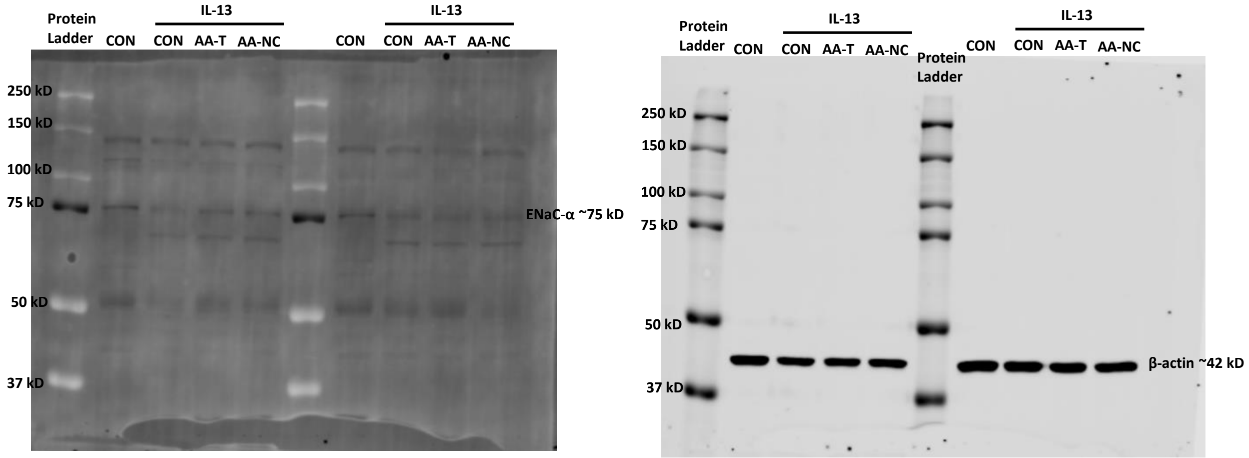

ENaC-α Blot 10

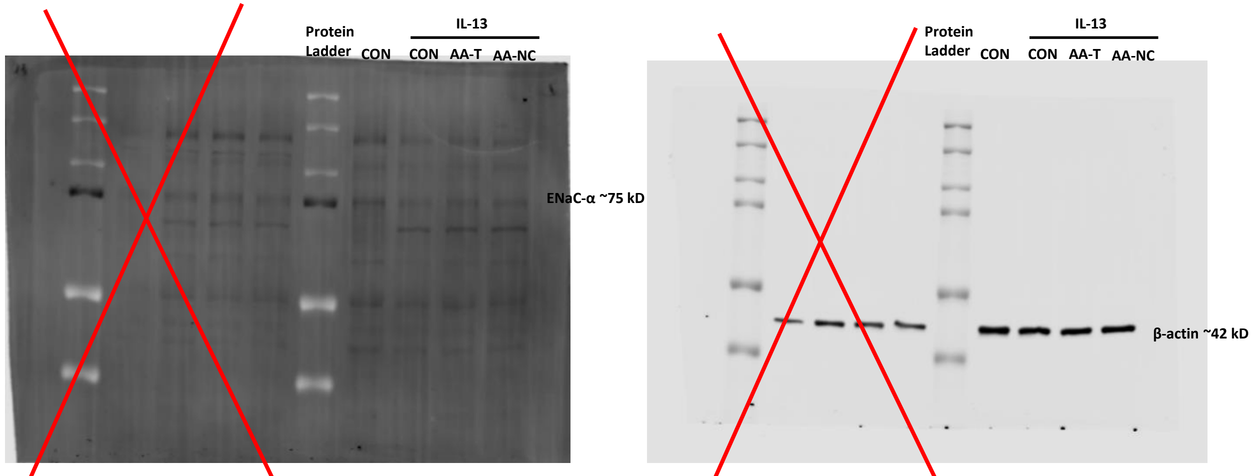

ENaC-β Western blot images

ENaC-β Blot 1

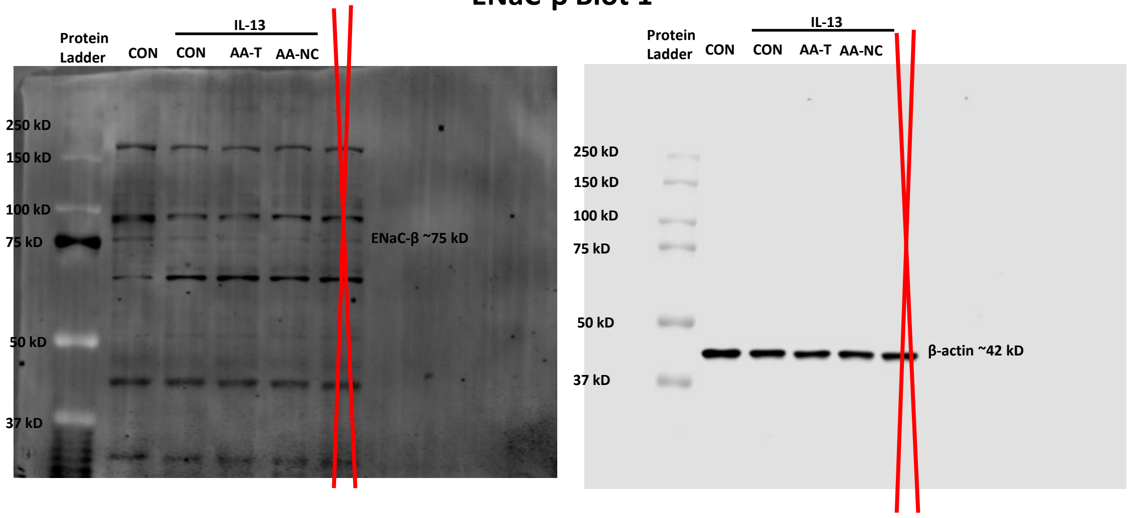

ENaC-β Blot 2 & 3

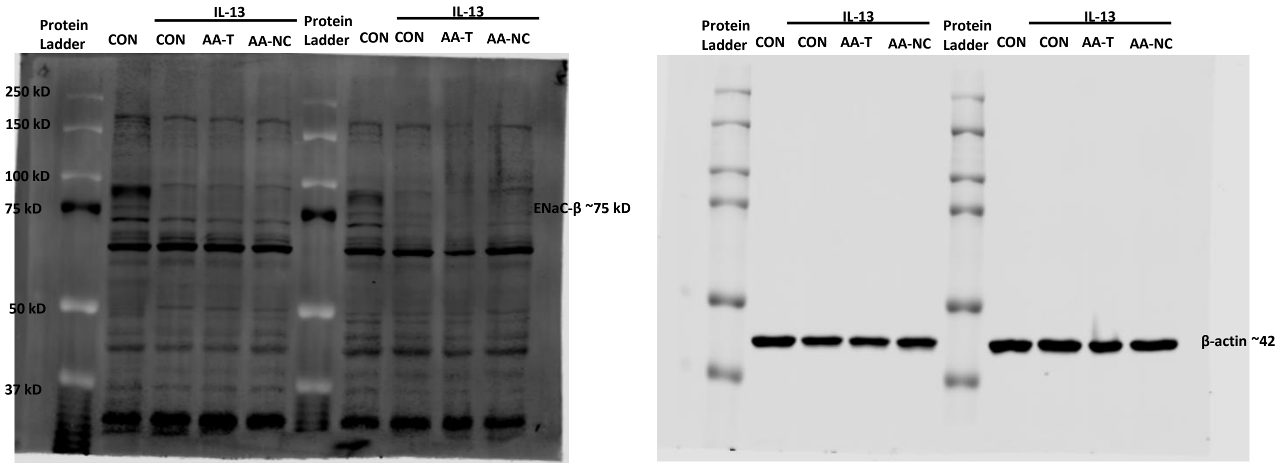

ENaC-β Blot 4

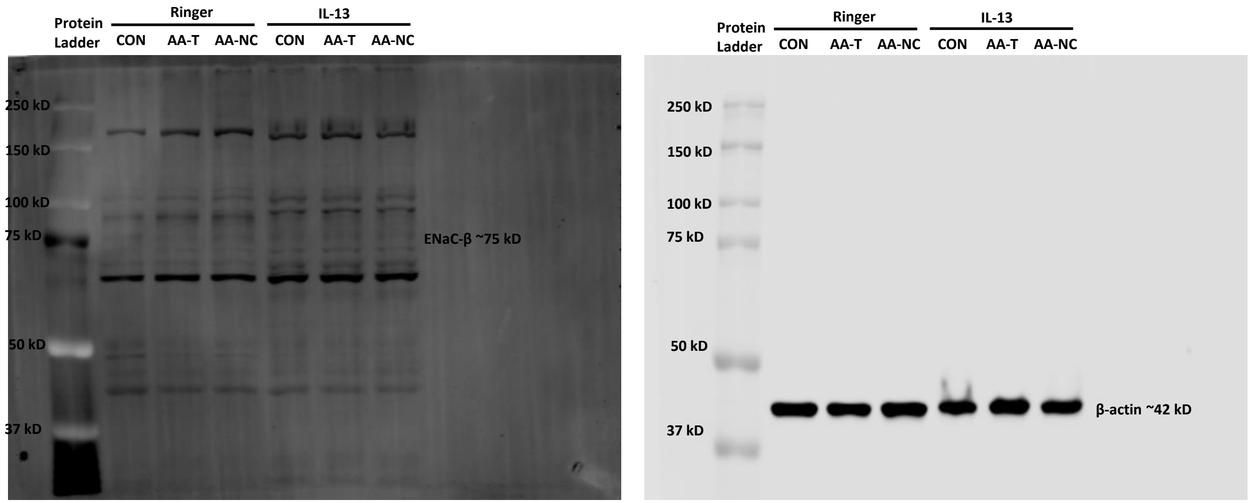

ENaC-β Blot 5

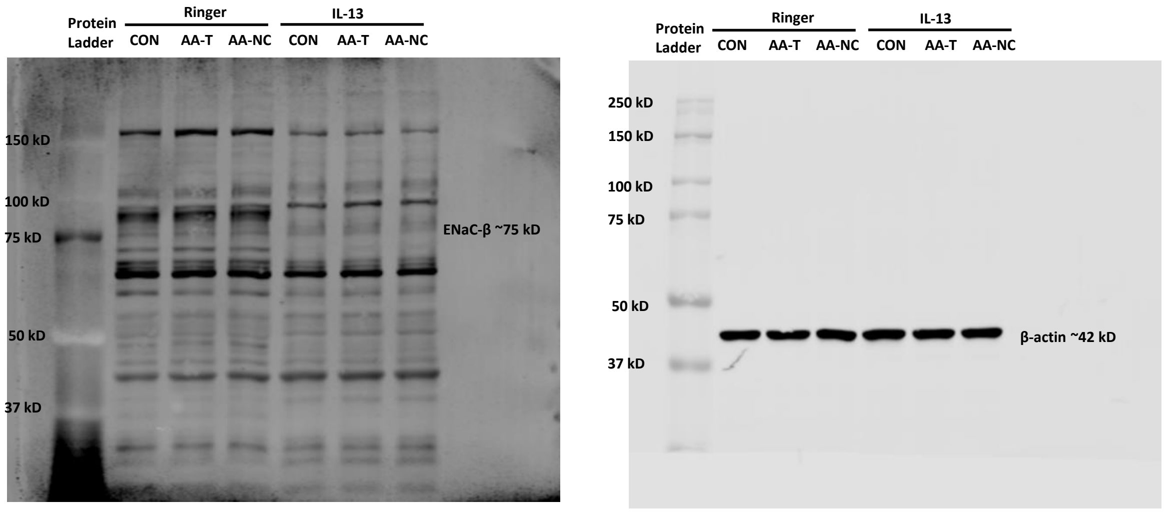

ENaC-β Western blot images

ENaC-β Blot 6

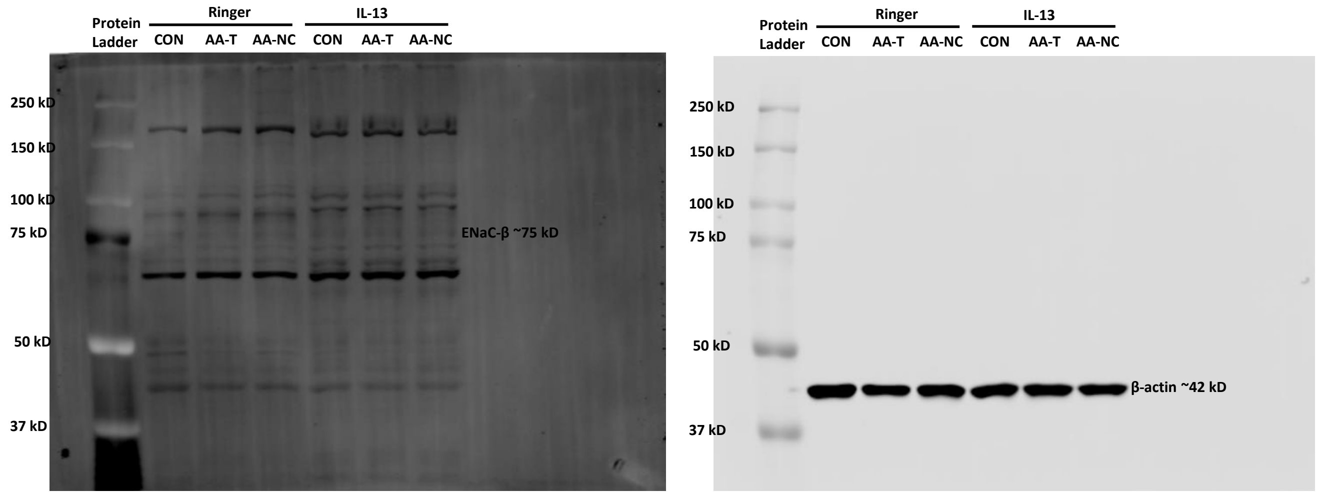

ENaC-β Blot 7

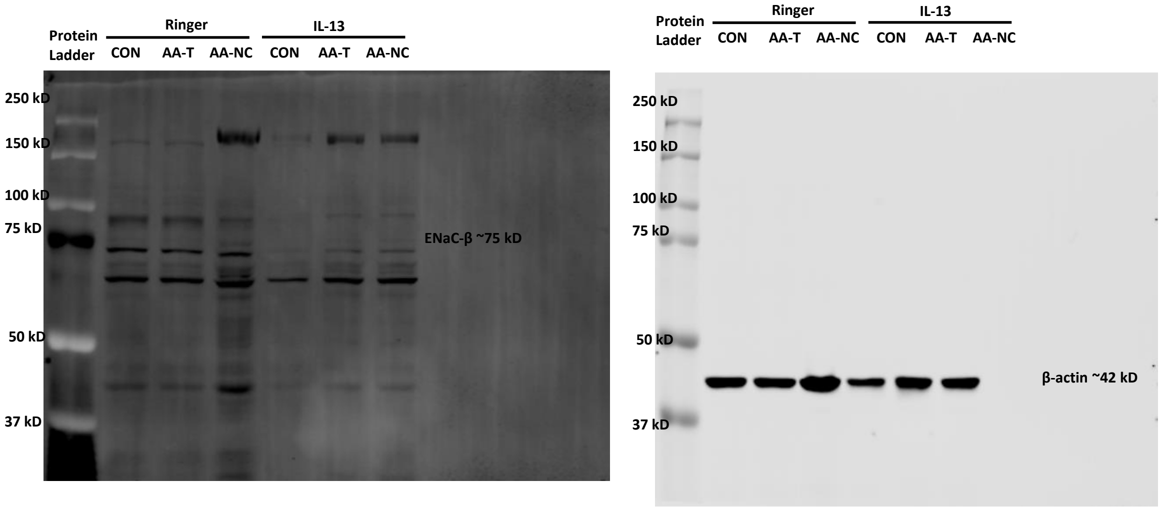

ENaC-β Blot 8

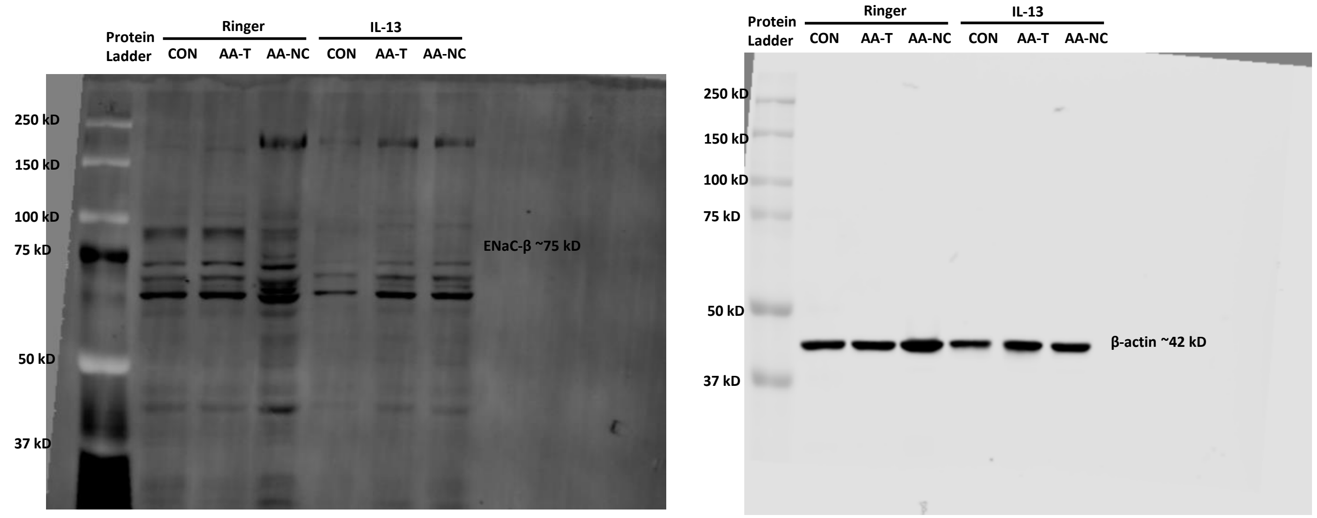

ENaC-γ western blot images

ENaC-ϒ Blot 1

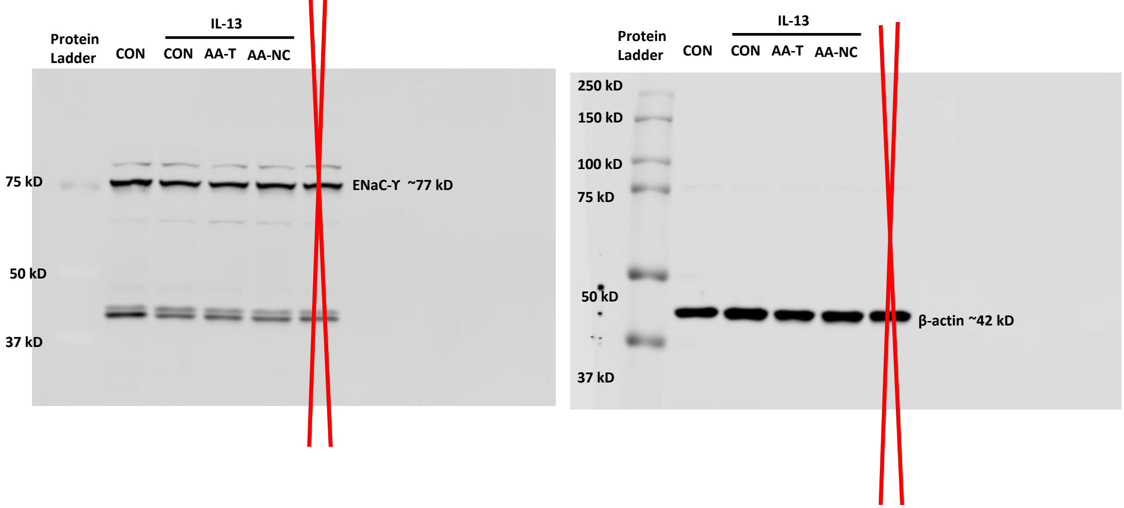

ENaC-ϒ Blot 2

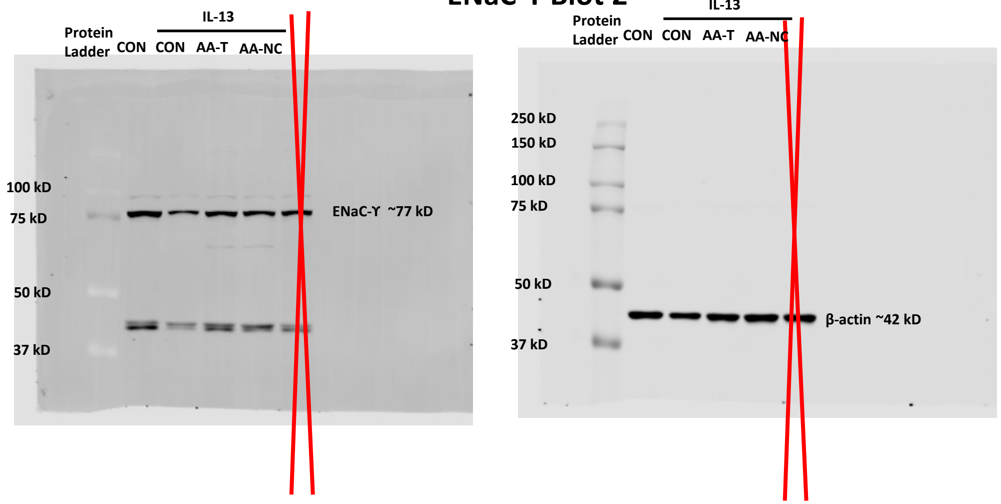

ENaC-ϒ Blot 3

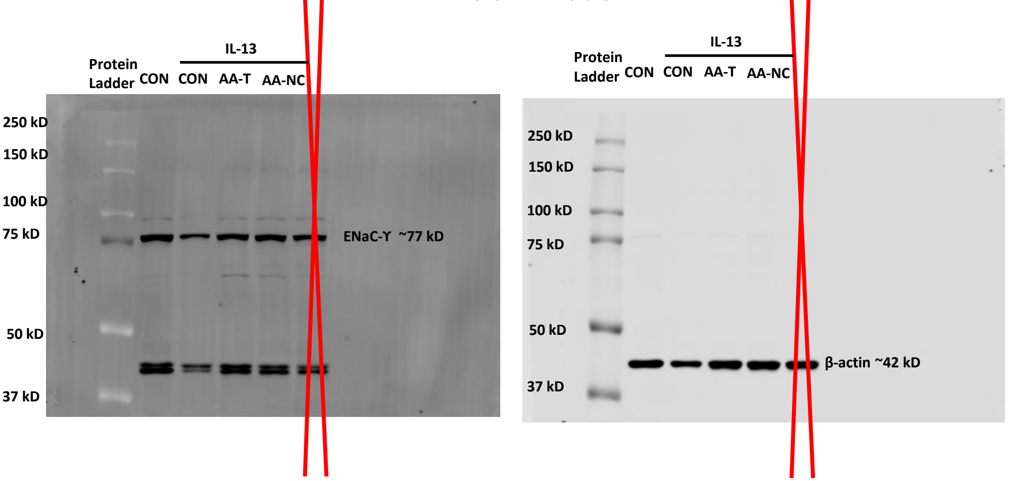

ENaC-ϒ Blot 4

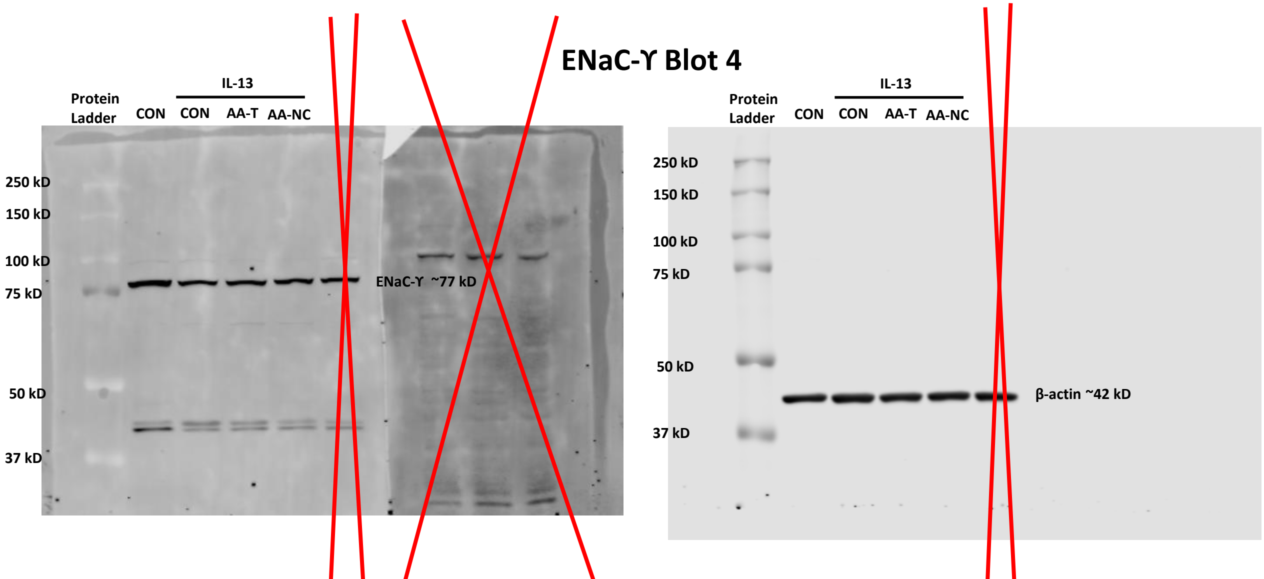

ENaC-γ western blot images

ENaC-Ɣ Blot 5

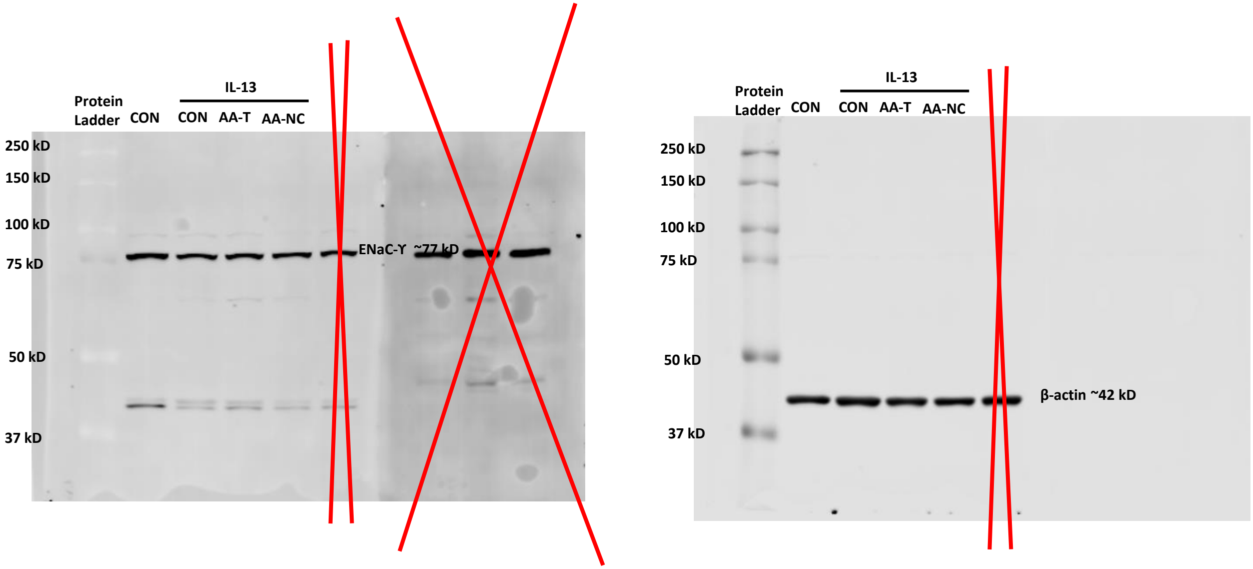

ENaC-Ɣ Blot 6

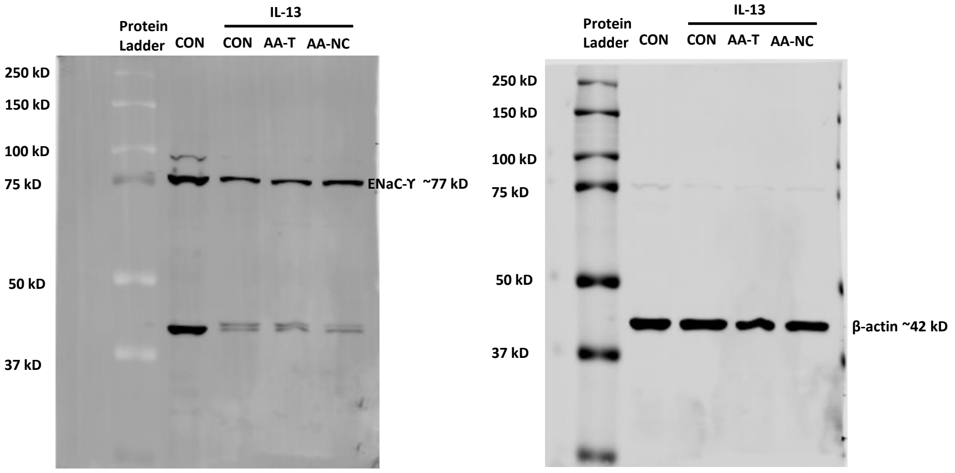

ENaC-Ɣ Blot 7

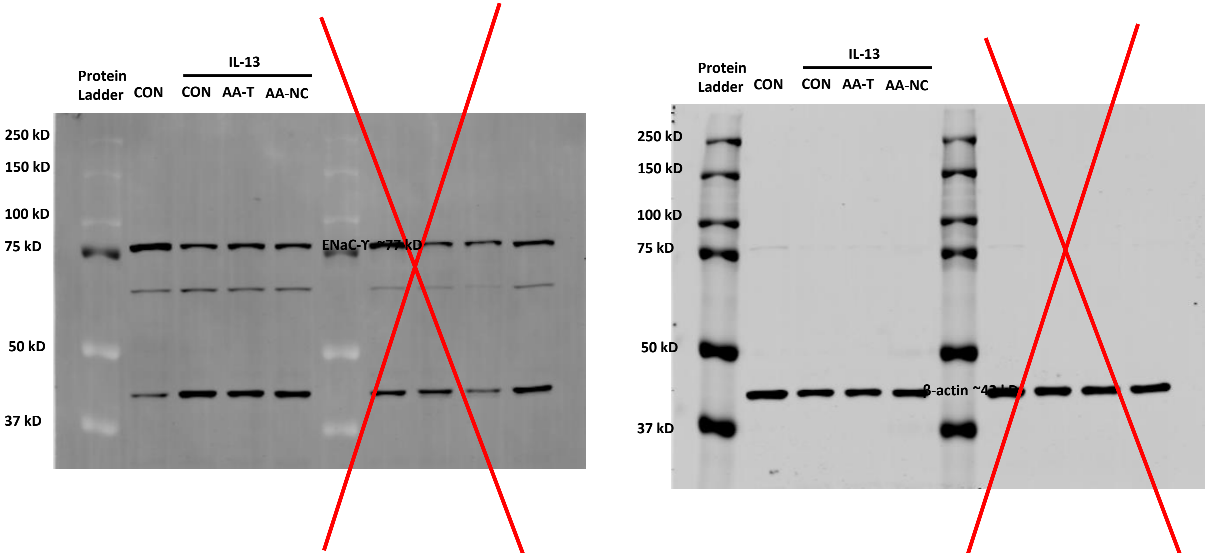

ENaC-Ɣ Blot 8

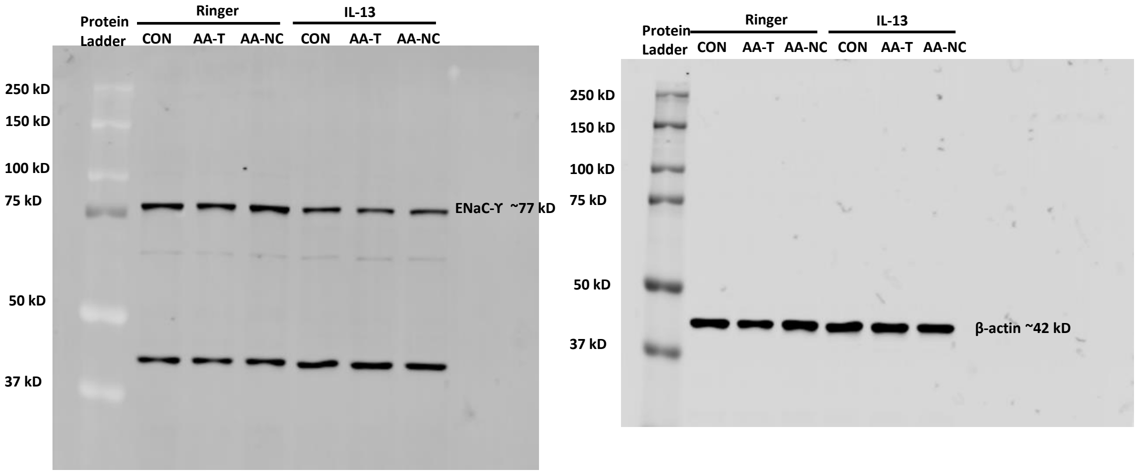

ENaC-γ western blot images

ENaC-ϒ Blot 9

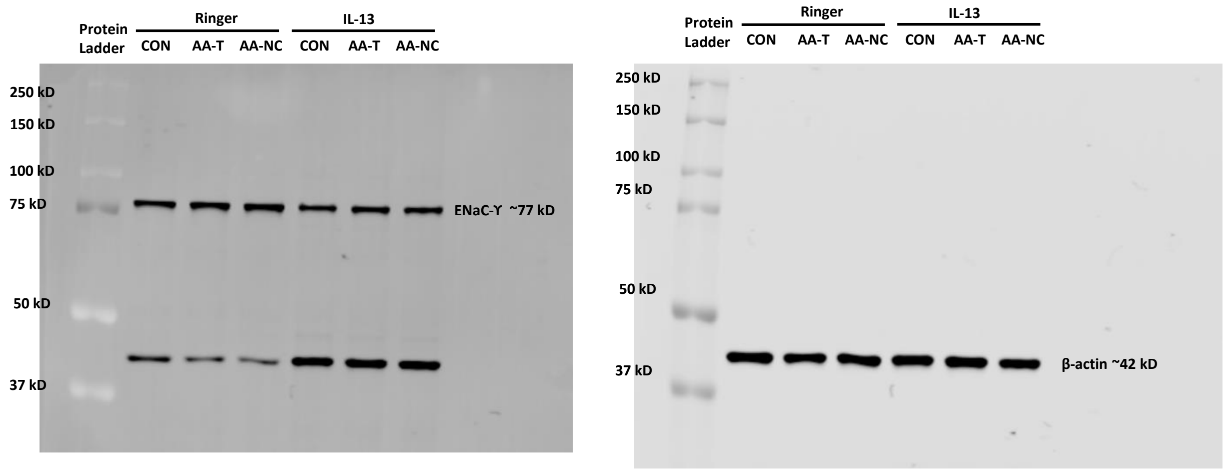

ENaC-ϒ Blot 10

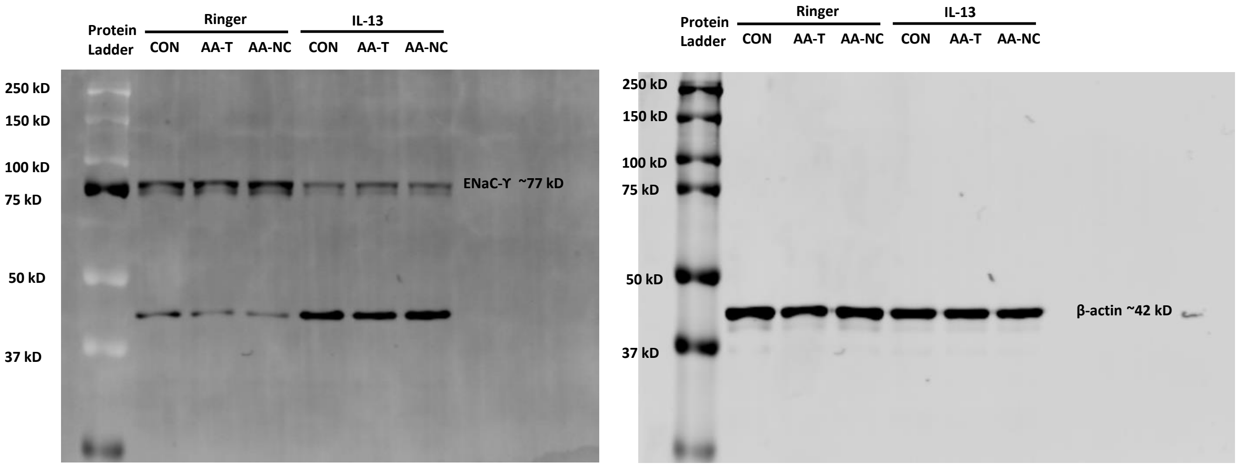

ENaC-ϒ Blot 11

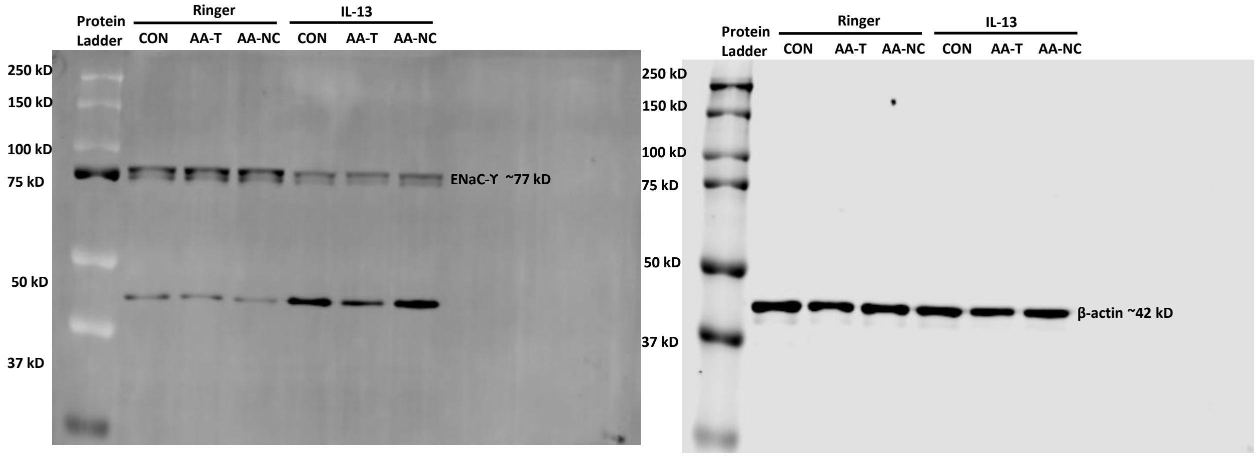

ENaC-ϒ Blot 12

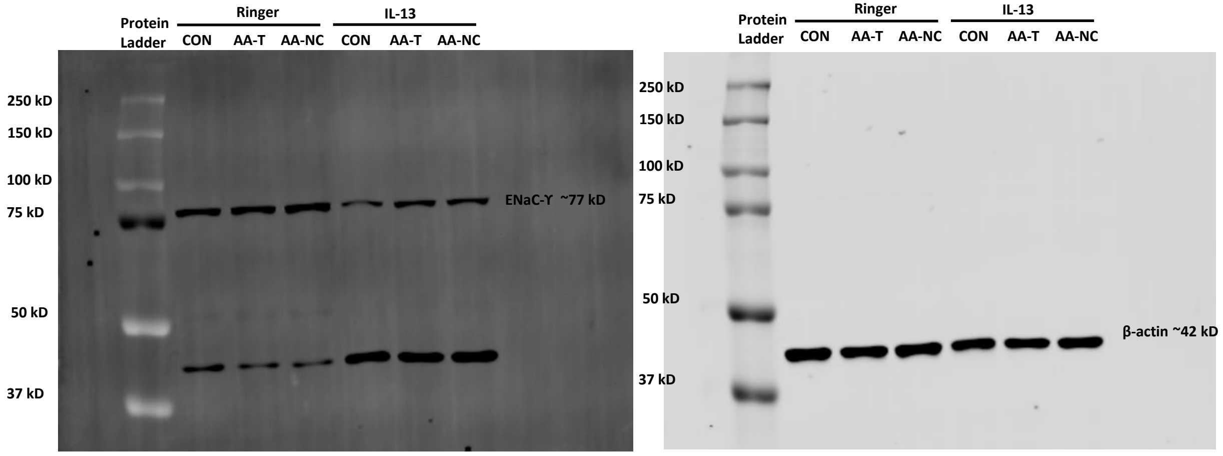

ENaC-γ western blot images

ENaC-ϒ Blot 13

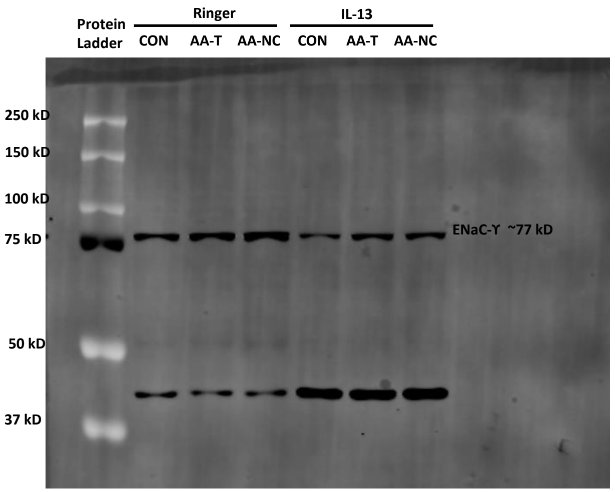

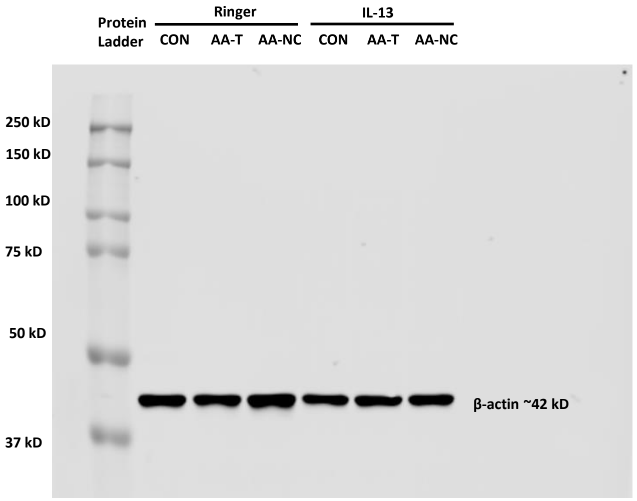

Supplement: S1 Raw images — (PDF) [file pone.0307809.s001.pdf]
